# Decoding the Molecular Drivers of Epithelial to Mesenchymal Transition in Breast Cancer: Insights into Epithelial Plasticity and Microenvironment Crosstalk

**DOI:** 10.3390/biology15030265

**Published:** 2026-02-01

**Authors:** Emanuela Peri, Miriam Buttacavoli, Elena Roz, Ida Pucci-Minafra, Salvatore Feo, Patrizia Cancemi

**Affiliations:** 1 Department of Biological Chemical and Pharmaceutical Sciences and Technologies (STEBICEF), University of Palermo, 90128 Palermo, Italy; emanuela.peri@unipa.it (E.P.); miriam.buttacavoli@unipa.it (M.B.); salvatore.feo@unipa.it (S.F.); 2 La Maddalena Hospital III Level Oncological Department, Via San Lorenzo Colli, 312, 90145 Palermo, Italy; roz@lamaddalenanet.it; 3 Experimental Center of Onco Biology (COBS), 90145 Palermo, Italy; pucci.ida@gmail.com

**Keywords:** breast cancer, epithelial-to-mesenchymal transition, proteomics, data mining, epithelial plasticity, cancer stem cells, proteoglycans

## Abstract

Recent evidence indicates that both epithelial-to-mesenchymal transition (EMT) and its reverse process, mesenchymal-to-epithelial transition (MET), are key mechanisms driving breast cancer (BC) metastasis. During EMT, epithelial BC cells acquire mesenchymal traits that enhance motility, invasiveness, and resistance to therapy. A deeper understanding of EMT regulation may therefore unveil novel therapeutic targets to limit disease progression. In this study, we analyzed the expression of key EMT-associated proteins, namely Vimentin, E-cadherin, Cytokeratin-18, and alpha-smooth muscle actin, in a cohort of 95 BC tissue samples and observed marked intra- and inter-tumoral heterogeneity. Notably, we found positive correlations between epithelial and mesenchymal markers, supporting the presence of hybrid epithelial/mesenchymal phenotypes and substantial cellular plasticity, which may contribute to BC heterogeneity. High heterogeneity in marker expression was also detected between tumor tissues and matched adjacent normal tissues. The unexpected complexity uncovered at the protein level prompted us to question whether single markers or limited proteomic panels are sufficient to capture the EMT landscape in BC. Through integrative bioinformatics, we defined a novel EMT gene signature significantly associated with prognosis. Functional enrichment revealed pathways related to extracellular matrix organization, proteoglycans, and intercellular communication, emphasizing the dynamic bidirectional crosstalk between BC cells and the tumor microenvironment. Moreover, we identified a gene cluster linked to cancer stem cell-like features, which may be clinically relevant for patient risk stratification. Overall, our findings underscore the complexity of EMT regulation in BC and introduce a new EMT signature with potential prognostic and therapeutic relevance.

## Simple Summary

Epithelial-to-mesenchymal transition (EMT) is a hallmark of cancer and plays a critical role in breast cancer progression. Here, we analyzed the expression of EMT-related markers, including Vimentin, E-cadherin, Cytokeratin-18, and α-SMA, in a cohort of 95 breast cancer tissue samples, revealing marked intra- and inter-tumoral heterogeneity. Positive correlations between epithelial and mesenchymal markers supported the presence of hybrid epithelial/mesenchymal phenotypes and high cellular plasticity. The investigation of the molecular basis of this plasticity was performed by integrative bioinformatics analyses, leading to the identification of a novel EMT gene signature significantly associated with prognosis. Functional enrichment analyses underscored the dynamic interplay between tumor cells and microenvironment. Moreover, a gene cluster associated with cancer stem cell-like features that may be clinically relevant for patient risk stratification was identified. Overall, our findings emphasize the complexity of EMT regulation in breast cancer and propose a new EMT signature with potential prognostic/therapeutic relevance.

## 1. Introduction

Breast cancer (BC) is the most common cancer worldwide, associated with a complex interplay of genetic, epigenetic, hormonal, and environmental factors. Approximately 90% of BC-related deaths result from local invasion and distant metastasis of tumor cells [1]. BC is a highly heterogeneous disease driven by molecular alterations in mammary epithelial cells, leading to different disease manifestations in individual patients. Along with these patient-to-patient differences, referred to as intertumoral heterogeneity, significant diversity can exist within tumor cell subpopulations of the same patient, referred to as intratumoral heterogeneity.

Epithelial-to-mesenchymal transition (EMT) represents a key biological program that underlies multiple cancer hallmarks, including tumor initiation, invasion, dissemination, and therapy resistance [2]. EMT involves a phenotypic shift whereby epithelial cells downregulate markers such as E-cadherin and cytokeratins while upregulating mesenchymal markers like Vimentin and N-cadherin. This transition leads to cytoskeletal remodeling, loss of cell polarity, and increased migratory and invasive capabilities [3]. Mesenchymal cells, degrading the basal lamina, invade the underlying connective tissue, thereby creating a new microenvironment that supports the survival and metastasis of cancer cells [4,5]. Cancer cells with mesenchymal and motile properties can migrate either individually (single-cell migration) or in clusters, demonstrating partial EMT characteristics (collective migration). However, EMT is not a binary event; instead, cells may occupy intermediate or “hybrid” states [6,7] reflecting epithelial–mesenchymal plasticity (EMP). This plasticity allows tumor cells to dynamically adapt to environmental cues, including signals from the metastatic niche or therapeutic pressure [5,8]. Notably, EMP has been linked to the acquisition of cancer stem cell (CSC)-like properties, enhancing tumor-initiating capacity, self-renewal, and multilineage differentiation. The integration of signals from the tumor microenvironment (TME), including hypoxia, immune infiltration, stromal interactions, and extracellular matrix (ECM) remodeling, further modulates EMT, and contributes to tumor progression [9]. Together, these aspects, along with the complex interplay between the genome, epigenome, transcriptome, proteome, and external microenvironmental components, explain both inter- and intra-tumoral heterogeneity [10]. Specifically, microenvironmental factors, such as tumor hypoxia, crosstalk with the surrounding tumor stroma (including endothelial cells, pericytes, and fibroblasts), and the involvement of various immune cells from both the innate and adaptive immune systems, trigger a bidirectional interaction that leads to unpredictable and dynamic changes during cancer progression, both in lymph node [11] and distant metastasis.

The EMT contributes to nearly all the hallmarks of cancer, and its role in tumorigenesis has been demonstrated in various cancers, including BC. Despite its complexity, due to context-dependent EMT mechanisms, a comprehensive understanding of this process remains challenging.

In this study, we aimed to dissect the molecular features underlying EMT in BC through a combination of proteomic and bioinformatics analyses. We analyzed canonical EMT markers, including Vimentin (VIM), alpha-smooth muscle actin (ACTA2 or α-SMA), Cytokeratin-18 (KRT18), and E-cadherin (CDH1) in BC and matched normal tissues. We identified marked interpatient heterogeneity, including the expression of multiple isoforms of Vimentin, E-cadherin, and Cytokeratin-18. Vimentin and α-SMA positively correlated with epithelial markers, supporting the existence of phenotypic plasticity and hybrid epithelial/mesenchymal states beyond the classical EMT model.

The unexpected complexity uncovered at the protein level prompted us to question whether single markers or limited proteomic panels are sufficient to capture the EMT landscape. To overcome these limitations, we integrated our experimental findings with a systematic in silico approach, using the EMTome database to retrieve a comprehensive set of EMT-associated genes relevant in breast cancer. Functional enrichment revealed strong links to extracellular matrix remodeling, proteoglycans, and tumor microenvironment interactions, highlighting bidirectional crosstalk between tumor cells and their niche. Notably, the EMT signature overlapped with cancer stem cell biomarkers, underscoring the tight interplay between EMT, stemness, and the tumor microenvironment, probably responsible for cellular plasticity. Overall, our findings reveal a complex interplay between EMT, the tumor microenvironment, and CSCs, underscoring the need for refined stratification strategies in BC management. Functional studies represent important future directions to strengthen the biological and clinical relevance of our findings.

## 2. Materials and Methods

### 2.1. Tissue Samples from BC Patients

In this study, 95 breast tissue samples from patients diagnosed with BC were used. The clinicopathological characteristics are summarized in Supplementary Table S1. All patients underwent surgical removal of the tumor at La Maddalena Hospital, without any cytotoxic treatment prior to surgery. Tumor specimens, containing at least 80% tumor cells, were snap-frozen and stored at −80 °C until protein extraction was performed. Protein extraction was also performed on 24 matched tumoral and normal adjacent tissues. The study was conducted in accordance with all required ethical standards, with the informed consent of patients and with the approval of the Institutional Review Board (N° 515/2008, 13 May 2008) from the La Maddalena Hospital, as previously reported [12,13]. The frozen tissues were homogenized in an ice bath with 50 mM Tris-HCl pH 7.5, 0.003% penicillin, 0.005% streptomycin, and then incubated overnight at 4 °C with rotation. Tissue lysates were centrifuged several times at 10,000 rpm for 20 min to remove cellular debris. The protein content in the tissue extracts was quantified using the Bradford assay.

### 2.2. Electrophoresis SDS-PAGE and Western Blotting

Aliquots containing 20 µg of cell lysates were separated by electrophoresis on 10% sodium dodecyl sulfate (SDS)–polyacrylamide gels, under reducing conditions, and electro-transferred into a nitrocellulose membrane for 1 h at 100 V and stained with Ponceau S (Sigma-Aldrich, St. Louis, MO, USA). Membranes were blocked with 5% milk in T-TBS solution 0.05% Tween-20 for 1 h at room temperature and then incubated overnight at 4 °C with a mouse monoclonal antibody for Vimentin (Santa Cruz Biotechnology, Dallas, TX, USA sc-6260), E-cadherin (Cell Signaling Technology, Danvers, MA, USA #14472), Cytokeratin-8 (Santa Cruz Biotechnology, Dallas, TX, USA sc-8020), Cytokeratin-18 (Santa Cruz Biotechnology, Dallas, TX, USA sc-51582), alpha-Smooth Muscle Actin (Sigma-Aldrich, St. Louis, MO, USA #A5228) or and β-actin (Santa Cruz Biotechnology, Dallas, TX, USA sc-47778). Following incubation with the mouse peroxidase-linked antibody (Promega, Fitchburg, WI, USA W402B or Invitrogen, Waltham, MA, USA #31430), the reaction was revealed by the ECL detection system, using ChemiDoc™ MP System (Bio-Rad, Milano, Italy). The correct protein loading was ascertained by red Ponceau staining and immunoblotting for β-actin. Bands quantification was performed using ImageJ software (version 1.53k). Western blot quantification was normalized against the β-actin signal. The correlation between epithelial and mesenchymal markers and clinicopathological characteristics was analyzed by Pearson’s test (Supplementary Table S2), as the variables analyzed are continuous and approximately normally distributed. A correlation coefficient was considered statistically significant when the *p* value was ≤0.05. All Western blot analyses were performed as technical replicates. To minimize technical variability, all patient samples were processed and analyzed in parallel blots, under identical conditions whenever possible, and when possible, membranes were stripped and reprocessed (Supplementary Figures S10–S19).

### 2.3. In Silico Bionformatic Analysis

EMTome (http://www.emtome.org/, accessed 20 June 2024) provides lists of genes associated with both EMT and MET across cancer types. It has a simple and intuitive user interface comprising four modules: EMT core signature, EMT-related gene retrieval, EMT-related gene interactome, and EMT-signature score. In this study, the EMT core signature module was used to identify a gene list specifically involved in the EMT of BC [14].

UALCAN database (http:///ualcan.path.uab.edu/, accessed 20 June 2024), contains expression data from 1097 patients with BC and 114 healthy patients, derived from the TCGA database. The expression levels of queried genes are normalized as transcripts per million reads. For each gene of interest, the database provides graphs and plots of the expression levels in normal and cancer tissues, displayed as box-whisker plots. Welch’s T-test estimated the significance of differences in expression levels between normal and primary tumors, and a *p* value ≤ 0.05 was considered as significant [15].

Kaplan–Meier plotter (http:///www.kmplot.com, accessed 20 June 2024), is an online database that correlates the expression levels of queried genes with survival data, evaluated as DMFS (Distant Metastasis Free Survival), OS (Overall Survival), and RFS (Relapse Free Survival, survival free from relapse) [16]. Survival graphs were obtained by splitting patients into two cohorts, based on gene expression levels (high expression versus low expression), selecting the optimal cut-off, and using the JetSet probe. When the optimal cut-off is selected, all possible cutoff values between the lower and upper quartiles are computed, and the best performing threshold is used as cut-off. Kaplan–Meier plotter applies appropriate statistical corrections for multiple testing as part of its standard workflow. Hazard ratios and correction for multiple testing are automatically provided by the software. In particular, Hazard ratios, and False Discovery Rate in addition to the *p*-value, are automatically calculated by the software.

The Tumor IMmune Estimation Resource (TIMER) database (https://cistrome.shinyapps.io/timer/, accessed 20 January 2026) is a comprehensive resource for systematic analysis of immune infiltrates across different cancer types [17]. It was applied to determine the relationship between EMT markers expression levels and immune infiltration based on the CIBERSORT algorithm. Among the tumor-infiltrating immune cells, the correlation between the expression level of EMT markers and immune infiltration, including cancer-associated fibroblasts, macrophages, endothelial cells, CD8^+^ T cells, and CD4^+^ T cells, was detected.

STRING (https:///string-db.org/, accessed 20 June 2024) is an online database that allows the study of direct (physical) and indirect (functional) protein–protein interactions derived from different sources (Genomic Context Predictions, High-throughput Lab Experiments, (Conserved) Co-Expression, Automated Textmining, Previous Knowledge in Databases, Coverage) [18].

Breast cancer Gene Expression Miner v4.5 database (bcGenExMiner v4.5), is an online statistical mining tool (http://bcgenex.ico.unicancer.fr, accessed 20 June 2024) of published annotated BC transcriptomic and RNA-seq data [19]. bcGenExMiner enables the exploration of gene expression, prognostic, and correlation analyses. The association between selected EMT-genes and grading (G1/G2/G3) was analyzed using the Gene expression-based Outcome for Breast Cancer Online (GOBO) database [20]. GOBO (http://co.bmc.lu.se/gobo, accessed 20 June 2024) contains an 1881-sample BC data set generated on Affymetrix U133A microarrays (Affymetrix, Inc., Santa Clara, CA, USA). The list of genes and protein abbreviations used in the text are reported in Supplementary Table S5.

## 3. Results

### 3.1. Proteomic Analysis of Vimentin, α-SMA, Cytokeratin-18 and E-Cadherin in BC Tissues

**Figure 1:**
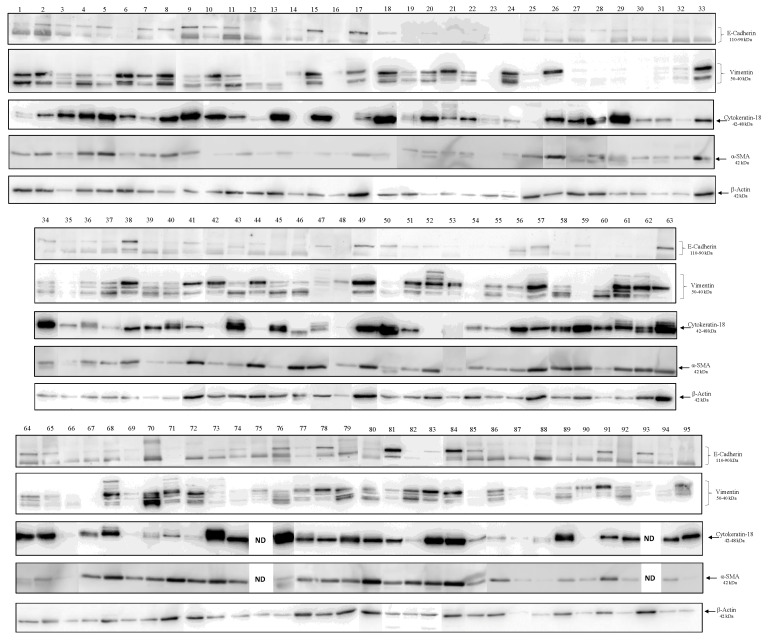
Western blot analysis performed on 95 BC tissue protein extracts to evaluate E-cadherin (110–90 kDa), Vimentin (50–40 kDa), Cytokeratin-18 (42–48 kDa) and α-SMA (42 kDa) expression. β-actin (42 kDa) was used as an internal control. ND = not detected. Each lane represents a tissue extract from an individual patient (Supplementary Figures S10–S14). Interestingly, a positive correlation between the expression levels of all detected Vimentin isoforms and both epithelial markers (Cytokeratin-18 and E-cadherin) was found (Figure 2). A positive correlation was also found between α-SMA and E-cadherin (Supplementary Figure S1, Supplementary Table S2). However, no correlation was observed between the epithelial markers themselves or between the mesenchymal markers. This interesting result, unexpected based on the classical EMT model, suggests that in BC cells, both epithelial and mesenchymal phenotypes coexist within the tumor mass, and reflect the activation of multiple and context-dependent mechanisms.

A proteomic analysis was performed on a cohort of 95 BC tissue samples to assess the expression levels of mesenchymal (Vimentin and α-SMA) and epithelial markers (Cytokeratin-18 and E-cadherin), by using Western blot (Figure 1). Detailed information on the clinical features of enrolled patients, including tumor grading and histology, was summarized in Supplementary Table S1. The E-cadherin antibody revealed two immunoreactive bands: one with a higher molecular weight (MW) of approximately 110 kDa, corresponding to the precursor of E-cadherin, not yet involved in cellular adhesion formation, and a lower MW band of about 90 kDa, corresponding to the mature form of the protein, likely engaged in active cellular adhesion. The antibody against Vimentin identified two main immunoreactive bands of MWs of 50 and 40 kDa and several minor bands ranging from 38 to 55 kDa, which probably represent different isoforms and/or proteolytic forms of the protein. The Cytokeratin-18 antibody revealed a main band of expected MW of approximately 42 kDa, and some isoforms ranging from 35 to 55 kDa, suggesting post translational modifications, such as phosphorylation and/or enzymatic mediated cleavage. The antibody against α-SMA revealed an immunoreactive band of the expected MW of about 42 kDa. Interestingly, a high degree of heterogeneity in the expression of all detected markers was observed in each patient, both qualitatively (e.g., presence of different isoforms) and quantitatively (e.g., band intensity). A limitation of this kind of analysis is the mentioned heterogeneity of BC tissues, so the density and number of neoplastic cells within the host stroma may influence the proteomic expression of analyzed proteins. To minimize this limitation and introduce an objective criterion for comparing protein expression of different tissues, the expression levels of individual proteins were normalized to β-actin levels in each tissue extract.

**Figure 3:**
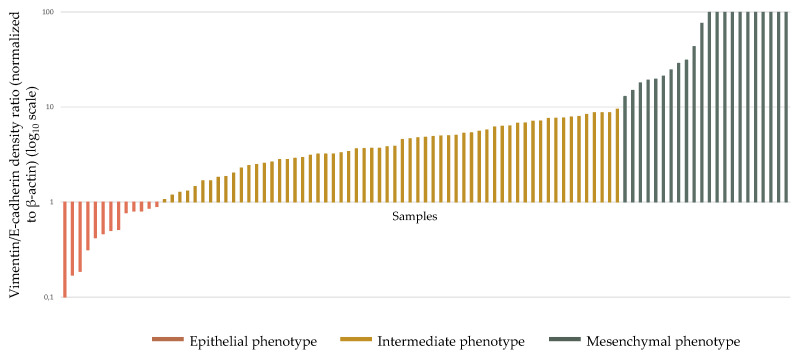
Vimentin/E-cadherin density ratio plotted on a log_10_ scale. A Vimentin/E-cadherin ratio < 1 (orange) indicates patients with a predominant epithelial phenotype; a ratio between 1 and 10 (yellow) indicates patients with an intermediate phenotype; and a ratio > 10 (green) indicates patients with a predominant mesenchymal phenotype. Patients are ordered according to increasing Vimentin/E-cadherin density ratio.

To evaluate the potential prevalence of the epithelial or mesenchymal phenotype between BC samples, the relationship between Vimentin and E-cadherin expression levels was investigated. A Vimentin/E-cadherin ratio < 1 would indicate a predominantly epithelial phenotype, while a higher ratio would suggest a predominantly mesenchymal phenotype. Interestingly, only a small fraction of samples (13/95, corresponding to 14% of patients) exhibited a Vimentin/E-cadherin ratio < 1. In contrast, 22 samples (23% of patients) displayed a Vimentin/E-cadherin ratio > 10. Most samples (60/95, corresponding to 63% of patients) showed an intermediate phenotype, with Vimentin/E-cadherin ratios ranging from 1 to 10 (Figure 3). A similar distribution pattern was observed evaluating the expression ratios of other epithelial and mesenchymal markers, such as α-SMA/E-cadherin (Supplementary Figure S2a) and Vimentin/Cytokeratin-18 (Supplementary Figure S2b). Although the expression patterns did not overlap perfectly, the prevalence of intermediate phenotypes remained consistent across the comparisons.

These findings indicate that the expression of epithelial and mesenchymal markers is not strictly mutually exclusive, and in the majority of BC tissues analyzed, the EMT is partial, characterized by a high degree of cellular plasticity, which is crucial for enabling cancer cells to adapt to changes in the tumor microenvironment. Moreover, epithelial plasticity can contribute to tumor heterogeneity, which refers to variations observed between tumors of the same type in different patients, as well as among cancer cells within a single tumor. The high heterogeneity of EMT markers expression pattern was also confirmed by limited correlation with clinicopathological characteristics, with no significant association, except for Ki-67, which was significantly correlated with both Vimentin and Cytokeratin-18 (Supplementary Table S2).

### 3.2. Proteomic Analysis of Vimentin, α-SMA, Cytokeratin-8, and E-Cadherin in BC Tissues and Matched Normal Tissues

**Figure 4:**
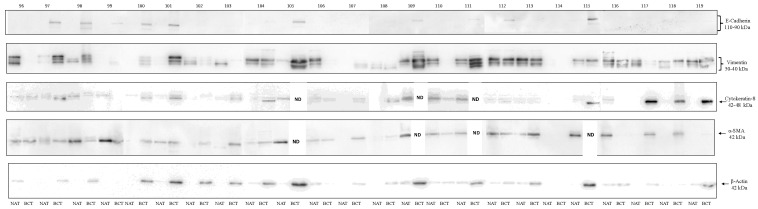
Western blot analysis performed on 24 surgical breast cancer tissues (BCT) and their matched normal adjacent tissues (NAT) to evaluate E-cadherin (110–90 kDa), Vimentin (50–40 kDa), Cytokeratin-8 (42–48 kDa), α-SMA (42 kDa), and β-actin (42 kDa) expression. ND = not detected (Supplementary Figures S15–S19).

**Figure 5:**
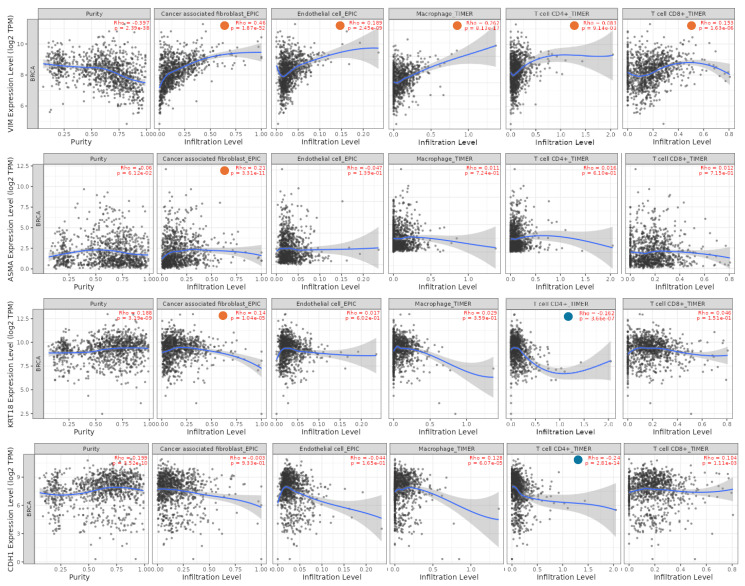
Correlation analysis between VIM (Vimentin), ACTA2 (α-SMA), KRT18 (Cytokeratin-18), and CDH1 (E-cadherin) expression and immune cell infiltration, including cancer-associated fibroblasts, macrophages, endothelial cells, CD8^+^ T cells, and CD4^+^ T cells, using the TIMER database. Orange circles indicate significant positive correlations, whereas blue circles indicate significant negative correlations. Each panel represents a scatterplot depicting the correlation between gene expression levels and the estimated abundance of the indicated immune or stromal cell population.

The proteomic analysis was also extended to a cohort of 24 surgical BC specimens and their matched adjacent normal tissues, collected from patients diagnosed with infiltrating ductal carcinoma of histological grade G2/G3. As shown in Figure 4, a marked heterogeneity was observed between tumoral tissues and their corresponding normal counterparts. Notably, in some tissues, no detectable signal was observed for certain markers. In several cases, EMT markers were expressed at comparable levels in both tissue types, or even more or less abundant in the normal tissue. Overall, epithelial markers such as E-cadherin and Cytokeratin-8, as well as β-actin, displayed low expression levels in normal tissues. In contrast, mesenchymal markers like Vimentin and α-SMA exhibited a more uniform distribution across both normal and tumoral samples. These findings open new interpretative scenarios regarding the dynamics of EMT during neoplastic transformation and highlight additional levels of complexity. A crucial aspect to consider is the specific cell type expressing each marker. Indeed, infiltration of stromal cells from the tumor microenvironment into the adjacent normal tissues may contribute to the observed expression patterns, and potentially explain the heterogeneity and partial overlap. An alternative explanation is that early molecular alterations are already occurring within histologically normal adjacent tissues, possibly triggered by initial transformation events or paracrine signals from the tumor microenvironment. Spatial localizion of these markers within tissue sections, using immunohistochemistry (IHC), could offer valuable insights into the cellular origin and microenvironmental context of these changes. However, complementary in silico analyses may further elucidateo the potential cellular contributors to the expression patterns detected in tissue samples. In this context, the relationship between EMT marker expression and immune cell infiltration, including cancer-associated fibroblasts, macrophages, endothelial cells, CD8^+^ T cells, and CD4^+^ T cells, was investigated using the TIMER database. This approach allows inference of cell-type-specific contributions based on correlation analyses across large transcriptomic datasets. The results revealed significant positive correlations between VIM (Vimentin) expression and multiple components of the immune microenvironment (Figure 5), supporting the notion that Vimentin signals detected in bulk tissues may, at least in part, derive from stromal and immune cell populations. As expected, ACTA2 (α-SMA) expression showed a strong positive correlation with cancer-associated fibroblast infiltration, further reinforcing its established role as a CAFs-associated marker. In contrast, CDH1 (E-cadherin) and KRT18 (Cytokeratin-18) exhibited only weak correlations with immune cell infiltration, consistent with their predominantly epithelial expression profiles.

### 3.3. In Silico Analysis

Proteomic results disclosed novel levels of complexity, including the presence of distinct isoforms with patient-specific expression patterns, suggesting that the regulatory landscape of EMT is more complex than previously assumed, and is responsible for cellular plasticity in BC. To overcome the limitations of single-protein analyses, we complemented our experimental data with a systematic bioinformatic approach to efficiently retrieve a comprehensive set of genes previously associated with EMT, thereby enabling a fully exploration of EMT-related signatures in breast cancer. The EMTome database was used to generate a gene list explicitly associated with breast EMT, by using the 14 studies recorded in the database (Supplementary Table S3). To strengthen the analyses, only genes identified in at least two independent studies were included. Applying this criterion, 144 breast EMT-related genes were selected (Supplementary Table S4). The obtained gene list was then utilized to explore the diagnostic and prognostic implications in BC. Firstly, the expression profile of each gene was evaluated between BC and its healthy counterpart by using the UALCAN database.

Among the 144 genes tested, 117 were significantly deregulated in BC tissues compared to the corresponding normal tissues (Table 1). In particular, 57 genes were upregulated (in red), while 60 genes were downregulated (in black) in BC compared to normal tissues, suggesting that EMT requires the coordinated activation and suppression of related genes (Supplementary Figure S3).

**Figure 6:**
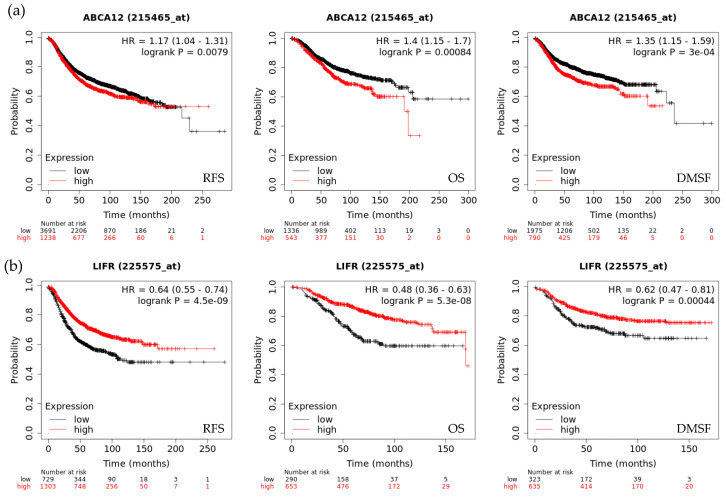
Survival analysis performed using KM plotter database. (**a**) Expression levels of ABCA12 were associated with worse prognosis across all the survival outcomes (evaluated as RSF, OS, and DMSF) associated. (**b**) Expression levels of LIFR were associated with better prognosis across all the survival outcomes (evaluated as RSF, OS, and DMSF).

The prognostic value of genes selected by EMTome was analyzed in BC using the Kaplan–Meier Plotter database (KM Plotter). KM Plotter correlates gene expression data with survival outcome, evaluated as RFS (Relapse Free Survival, *n* = 4929), OS (Overall Survival, *n* = 1879), and DMFS (Distant Metastasis Free Survival, *n* = 2765). For each gene, by selecting the best cut-off mode, the software splits patients into two groups (high and low expression) based on the most significant probe expression value, and returns a survival graph, with a log-rank value (Figure 6). The Logrank *p* values less than 0.05 were considered significant.

The expression levels of genes were considered significantly associated with prognosis when all survival outcomes, evaluated as RSF, OS, and DMSF, were coherently significant (Supplementary Figures S4–S9). In particular, the increased expression levels were significantly associated with a worse prognosis for 32 genes and significantly associated with a better prognosis for 28 genes (Table 2).

### 3.4. Biological Connectivity Between the EMT-Selected Genes

**Figure 7:**
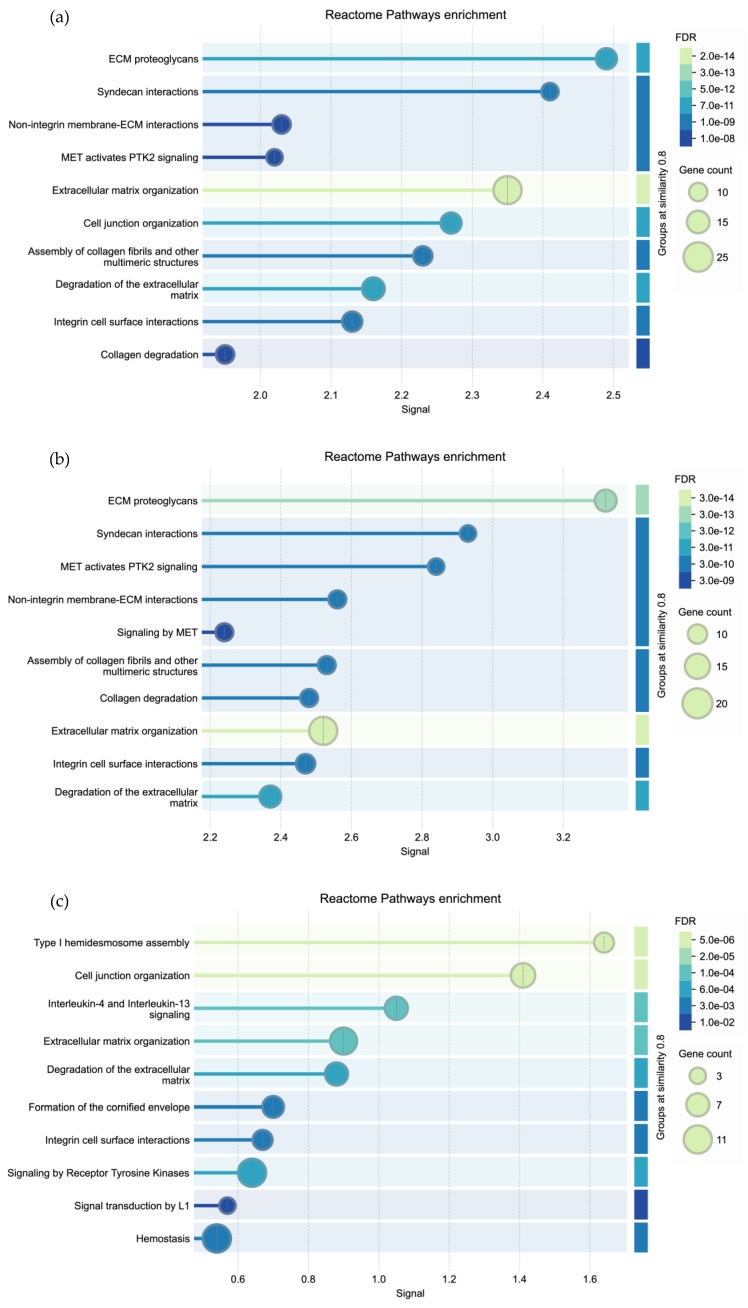
Reactome pathways enrichment analysis obtained by the STRING database using: (**a**) Genes found differentially expressed by UALCAN and significantly associated with prognosis by KM Plotter analysis (n = 129); (**b**) Genes upregulated in BC compared to normal tissues and genes whose elevated expression associated with worse prognosis (n = 67); (**c**) Genes downregulated in BC compared to normal tissues and genes whose reduced expression associated with a better prognosis (n = 73 genes).

Interpreting the obtained results to correlate genes with specific pathways is a challenging task. With this aim, the genes, whose expression levels were found to be significant in both KM Plotter and UALCAN databases, were further analyzed using STRING database, to verify if they act in a coordinated manner, possibly because they are part of the same biological pathway. In STRING, the predicted relationships between genes are summarized as a biological network, where proteins are represented as nodes and each relationship is represented as an edge between two proteins (or nodes). This network may then be explored to check the underlying enrichment pathways. When all significant genes identified by UALCAN and KM Plotter were considered (n = 129 genes) (Figure 7a), significantly enriched pathways included ECM proteoglycans, with a particular focus on Syndecan, as well as ECM organization and remodeling, and cell–cell and cell–matrix interactions. Collectively, the obtained results suggest that the identified genes are part of a network that could regulate or enable the transition from an epithelial to a mesenchymal phenotype, as well as its reverse process. Interestingly, the TME and EMT have a significant impact on tumor cell growth, metastasis, and therapeutic response, with complex interactions between cellular and non-cellular components of TME through the secretion of cytokines, growth factors, and matrix metalloproteinases (MMPs). When the gene list was divided based on the levels of upregulation/downregulation observed in both UALCAN and KM plotter, two distinct groups of genes were obtained, each providing valuable insights. The first group, consisting of genes upregulated in BC compared to normal tissues, and genes whose elevated expression was associated with a worse prognosis (n = 67 genes), confirmed that ECM proteoglycans like Syndecan and ECM remodeling are crucial in EMT, as they prepare cells to migrate and invade (Figure 7b).

The second group consisted of genes downregulated in BC compared to normal tissues, and genes whose reduced expression was associated with a better prognosis (n = 73 genes). In this case, pathway enrichment analysis (Figure 7c) revealed significant associations with Interleukin (IL)-4 and IL-13 signaling, tyrosine kinase receptor-mediated signaling, and L1 receptor signaling, as well as regulation of cell junctions and hemidesmosomes organization. Downregulation of these genes may lead to a more stable epithelial phenotype, potentially inhibiting the induction of EMT and thus contributing to a better prognosis.

To identify a specific EMT BC gene signature capable of distinguishing homogeneous patient groups, only overlapping results according to UALCAN (diagnostic significance) and KM plotter (prognostic significance) were considered.

**Figure 8:**
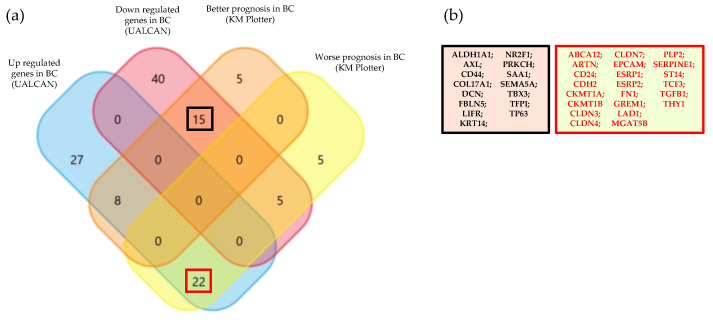
(**a**) Venn diagram between the gene surveys of UALCAN and Kaplan–Meier plotter. (**b**) The list of 37 genes, downregulated (black) and overexpressed (red), whose expression was found to be significant in both Kaplan–Meier plotter and UALCAN.

A concordance between UALCAN and KM Plotter results was found for 37 genes (Figure 8).

Specifically, 22 of these genes were overexpressed in tumor tissues compared to healthy tissues, and their overexpression was associated with a worse prognosis. By focusing on this subset of genes, it is possible to identify potential markers that are not only biologically relevant, but also crucial for understanding cancer progression and predicting patient outcomes. The remaining 15 genes were downregulated in the tumor tissue compared to healthy tissue, and their downregulation was associated with a better prognosis. These genes, when suppressed in BC, might contribute to a more favorable disease course and better survival rates, suggesting that their reduced expression could potentially play a protective role in preventing cancer progression or metastasis. For example, COL17A1, which is more expressed in healthy tissues, is shown to be related to the suppression of cell migration and invasion. In BC patients, high expression of COL17A1 is associated with a better prognosis [21]. The loss of LIFR has been correlated with the clinical progression of BC, and patients with stage III and IV cancer have a lower level of protein expression than patients with stage I and stage II cancer [22]. Additionally, FBLN5 exhibits anti-tumor potential in BC cells [23].

Interestingly, among these 37 genes, there are several key players in promoting stemness, suggesting that heterogeneous signals from the TME fuel CSCs, whose plasticity may be responsible for the detected hybrid TME [24]. For instance, TGF-β is one of the master players of stemness and EMT [25], while AXL, CD44, CD24, ALDH1A1, COL17A1, EPCAM, FN1, and claudins are biomarkers of BC stem cells [7,26,27,28,29,30,31].

To further explore the clinical relevance of these findings, their relationship with current clinical-pathological parameters, including the immunohistochemical expression of estrogen receptor (ER), progesterone receptor (PR), human epidermal growth factor receptor 2 (HER2), lymph node (N) metastasis status, and tumor grading (G1–G3) was assessed using the bcGeneXMiner and Gene expression-based Outcome for Breast Cancer Online (GOBO) databases (Table 3).

Consistent with our hypothesis, the 22 genes associated with poor prognosis (highlighted in red in Table 3) showed significant correlations with more aggressive clinical features, including ER−/PR−, HER2+, N+, and G3 tumors. These tumor characteristics are known to identify more aggressive forms of BC. In contrast, the 15 genes associated with a good prognosis (highlighted in black in Table 3) exhibited an inverse trend, correlating with ER+/PR+, HER2−, N−, and G1 tumors, which are typically less aggressive.

These findings reinforce the potential of this gene signature in predicting clinical outcomes based on tumor aggressiveness and molecular characteristics.

## 4. Discussion

EMT and its reverse process, MET, are fundamental drivers of breast cancer progression, metastasis, and therapeutic resistance [32,33,34]. By enabling tumor cells to transition between epithelial and mesenchymal states, these processes confer phenotypic plasticity that fuels tumor heterogeneity and metastatic potential [7]. Despite extensive investigation, the molecular mechanisms regulating EMT within breast tumors remain incompletely understood.

In this study, we combined proteomic and in silico analyses to characterize the EMT landscape in BC, focusing on the expression of canonical EMT markers (Vimentin, E-cadherin, Cytokeratin-18, and α-SMA) and identifying an EMT-related gene signature with diagnostic and prognostic relevance. Proteomic profiling of 95 BC tissues revealed marked heterogeneity in the expression of selected markers. Multiple isoforms were detected for all proteins, except α-SMA, likely reflecting post-translational modifications or alternative splicing. Proteolytic processing of Vimentin and Cytokeratin-18 has been reported during apoptosis [35,36,37], while Vimentin isoforms have been linked to disease specificity in other cancers, such as renal carcinoma [38]. Our results pointed on specific roles of Vimentin isoforms that may contribute to different EMT states and tumor behaviors in BC. In fact, it was demonstrated that Vimentin expression could not differentiate between benign and invasive breast lesions, although its expression was correlated with tumor grade and associated with decreased survival in ductal carcinoma [39]. Notably, we observed positive correlations between all Vimentin isoforms and epithelial markers (Cytokeratin-18 and E-cadherin), as well as between α-SMA and E-cadherin. These findings challenge the classical binary EMT model and support a non-canonical EMT framework characterized by phenotypic plasticity and hybrid epithelial/mesenchymal states [6,40] that may sustain collective migration. Such co-expression likely reflects context-dependent EMT regulation driven by tumor microenvironmental signals, including cytokines such as TGF-β, which exerts dual tumor-suppressive and tumor-promoting roles depending on cellular context. Overall, our findings highlight the complexity of EMT regulation in BC and the need to account for EMT heterogeneity in biomarker studies. Quantification of the Vimentin/E-cadherin ratio revealed that a low percentage of BC samples displayed a predominantly epithelial phenotype or mesenchymal phenotype, while the majority exhibited an intermediate phenotype. The prevalence of intermediate states was further supported by additional epithelial/mesenchymal marker ratios, including α-SMA/E-cadherin and Vimentin/Cytokeratin-18. However, these combinations did not fully overlap, indicating marker-dependent variability in EMT. EMT is widely recognized as a non-binary process generating a continuum of hybrid epithelial/mesenchymal states [8], which are associated with enhanced stemness, phenotypic plasticity, tumorigenicity, drug resistance, and metastatic potential [41]. These states may exist as transient intermediates or stable phenotypes sustained by complex signaling and epigenetic networks [22,42,43], highlighting epithelial–mesenchymal plasticity (EMP) as a central feature of BC biology and suggesting the existence of numerous partial EMT states [44]. Consequently, EMT markers should be interpreted in a context-dependent manner. Although our BC tissues were enriched in tumor cells, we could not discriminate protein expression derived from distinct cellular compartments. This is particularly relevant given that a caspase-3–cleaved Vimentin variant secreted by tumor-associated macrophages correlates with tumor malignancy and lymph node metastasis [45]. Similarly, α-SMA expression has been extensively studied in cancer-associated fibroblasts [46], where it correlates with tumor progression, metastasis, prognosis, and overall survival [47], and more recently, in α-SMA–expressing breast cancer cells with variable effects on tumor growth, invasion, and recurrence [48]. Moreover, in normal breast tissue, α-SMA expression is restricted to myoepithelial cells, which act as a barrier to epithelial cell dissemination [49]. In silico analyses using TIMER provided valuable insights into the potential cellular contributors to the expression patterns detected in tissue samples. Given the variability of EMT responses in BC, a single mesenchymal gene expression program probably is insufficient to recapitulate the complexity of the process. To elucidate the transcriptional landscape of EMT, we queried the EMTome database and curated a list of 144 EMT-related genes specific to BC. Differential expression analysis via UALCAN identified 117 of these genes as significantly deregulated in tumor compared to normal tissues, suggesting a coordinated shift in gene networks during EMT. Among these, 57 genes were upregulated, and 60 were downregulated in BC. Prognostic assessment using KM Plotter showed that 32 genes were associated with poor prognosis, while 28 correlated with favorable survival outcomes. Identified genes are linked to the ECM and cell–matrix interaction, indicating a crosstalk between BC cells undergoing EMT and their surrounding tumor microenvironment (TME). Emerging evidence suggests that key features of the TME, such as immune cell infiltration, angiogenesis, and ECM composition, are closely correlated with patient survival and overall prognosis [50]. Finally, by cross-referencing both expression and prognostic data, we refined the EMT gene set to 37 candidates with robust clinical significance: the EMT-genes overexpressed in tumors and associated with poor prognosis correlated with aggressive clinical parameters (ER−, PR−, HER2+, nodal involvement, and high grade). In contrast, the downregulated genes were associated with more favorable features (ER+, PR+, HER2−, node-negative, low grade). Pathway enrichment analysis revealed that the upregulated genes, linked to poor prognosis, were enriched in ECM remodeling, proteoglycan pathways (notably Syndecan), and cell–matrix interactions. Generally, SDC2 is expressed at higher levels in mesenchymal cells, and its overexpression in epithelial-origin tumors seems to be correlated with aggressive behavior [51]. Conversely, the downregulated genes, associated with good prognosis, were enriched in Interleukin (IL)-4/IL-13 signaling, tyrosine kinase receptor pathways, and cell adhesion maintenance, suggesting their potential role in preserving epithelial integrity. Recently, IL-4 and IL-13 cytokines and their receptors have received significant attention as potential therapeutic targets in cancer, as their increased expression by immune and non-immune cells in the TME promotes tumor growth, survival, and immunosuppression [52]. Notably, the 37-gene signature overlaps extensively with cancer stem cell (CSC) markers, supporting the concept of stemness as a dynamic cellular state rather than a fixed hierarchy [53]. Several genes identified, such as CD44 [54], CD24 [55], ALDH1A1 [56], COL17A1 [57], EPCAM, FN1, AXL [58], CDH2, and claudins, are well-established CSC markers in BC [26,27,28,29,31,59,60]. Others regulate CSC proliferation or differentiation, including PRKCH [30], ST14 [61], and TCF3/TCF7L1 [62]. Conversely, ESRP1 and ESRP2 suppress CSC-associated phenotypes and proliferation [63,64], while LIF limits CSC tumorigenicity via Hippo signaling [65]. SAA1 [66] and GREM1 [67], are secreted by CSCs, and the latter also act as a TGF-β inhibitor and VEGFR2 ligand [68]. Moreover, several identified genes also support collective migration and invasion during EMT. CDH2 and PLP2 are essential for collective 3D migration and cytoskeletal remodeling [69,70], while SERPINE1 and SEMA5A promote migration, invasion, and angiogenesis [71]. MGAT5 regulates mechanotransduction-driven invasion in CSCs [72], whereas DCN acts as a suppressor of invasion and metastasis [73]. Additional surface markers such as Thy-1/CD90 [74] and LAD1 [75] further associate EMT with stemness and migratory capacity. Finally, CSCs are key contributors to therapy resistance. Claudins have been implicated in EMT-driven CSC enrichment and drug resistance [76]. Artemin (ARTN) promotes resistance to trastuzumab, chemotherapy, and radiation by inducing CSC-like traits [77,78], while ABCA12 supports CSC survival through lipid metabolism and chemoresistance [79]. TGF-β signaling, particularly through ECM components such as FN1, may further sustain CSC self-renewal by reinforcing stemness-associated signaling networks.

## 5. Conclusions

Our findings suggest that EMT in BC is not regulated by a canonical, linear, unidirectional process, but rather by a complex, reversible spectrum of phenotypic states modulated by the TME, extracellular signaling, and stemness, probably involving alternative EMT modalities, which necessarily deserve further functional and experimental validations. The proposed 37-gene EMT signature, derived from integrated bioinformatic and proteomic analyses, provides insights into the regulatory circuits underlying tumor plasticity. If validated in independent cohorts and through molecular, cellular, and spatially resolved approaches, this signature may contribute to patient stratification and support the development of diagnostic panels. Future studies using single-cell and spatial transcriptomic or IHC analyses will be critical to dissect how EMT, tumor–microenvironment interactions, and stemness programs are dynamically orchestrated within breast tumors and how they influence cancer progression and therapeutic response.

## Figures and Tables

**Figure 2:**
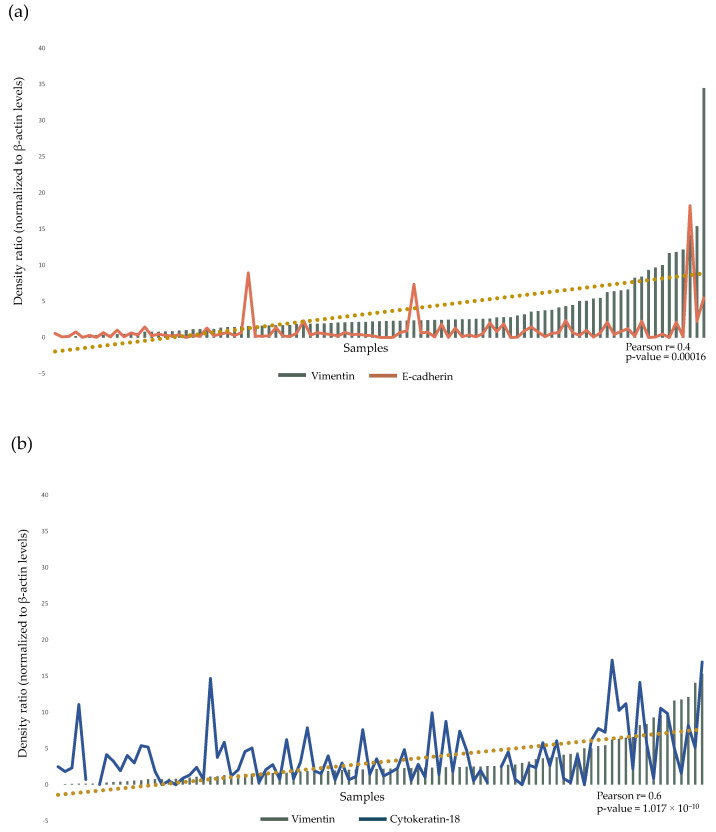
Histogram of E-cadherin and Vimentin (**a**) and Vimentin and Cytokeratin-18 (**b**) expression levels, normalized to β-actin levels, in the cohort of 95 BC tissues analyzed. Patients were sorted by increased levels of Vimentin expression. The dashed line shows the trend of the positive correlation between the selected markers.

**Table 1:** Genes significantly upregulated (in red) and downregulated (in black) in patients with BC vs. healthy subjects analyzed in UALCAN database, sorted by protein name.

Genes Upregulated in BC	Statistical Significance	Genes Upregulated in BC	Statistical Significance	Genes Downregulated in BC	Statistical Significance	Genes Downregulated in BC	Statistical Significance
ABCA12	6.03 × 10^−5^	KRT18	1.62 × 10^−12^	ABCC4	1.06 × 10^−3^	KRT17	5.55 × 10^−16^
ARTN	1.62 × 10^−12^	LAD1	1.62 × 10^−12^	ABLIM1	1.62 × 10^−12^	KRT5	<1 × 10^−12^
CD24	1.62 × 10^−12^	LRRC1	1.11 × 10^−16^	ACSL4	1.62 × 10^−12^	LAMA3	1.62 × 10^−12^
CDH1	9.56 × 10^−8^	MAL2	1.62 × 10^−12^	ADAM9	1.27 × 10^−8^	LIFR	<1 × 10^−12^
CDH2	3.45 × 10^−8^	MGAT5B	1.11 × 10^−16^	ALDH1A1	<1 × 10^−12^	MCAM	<1 × 10^−12^
CKMT1A	<1 × 10^−12^	MLPH	1.62 × 10^−12^	ALDH1A3	1.62 × 10^−12^	NR2F1	7.51 × 10^−9^
CKMT1B	1.62 × 10^−12^	MMP3	<1 × 10^−12^	ANXA8	3.67 × 10 ^−6^	NRP1	<1 × 10^−12^
CLDN3	<1 × 10^−12^	MMP9	2.09 × 10^−5^	AXL	3.33 × 10 ^−16^	PMP22	1.11 × 10^−16^
CLDN4	2.92 × 10^−8^	MST1R	<1 × 10^−12^	CD44	9.79 × 10^−5^	PRKCH	<1 × 10^−12^
CLDN7	1.62 × 10^−12^	OCLN	1.62 × 10^−12^	CDH3	1.10 × 10 ^−3^	PRRX1	4 × 10^−2^
COL1A1	1.62 × 10^−12^	PKP3	1.62 × 10^−12^	COL17A1	<1 × 10^−12^	PTX3	1.81 × 10^−8^
COL1A2	1.62 × 10^−12^	PLIN3	<1 × 10^−12^	DCBLD2	<1 × 10^−12^	RGL1	<1 × 10^−12^
COL3A1	<1 × 10^−12^	PLP2	1.62 × 10^−12^	DCN	1.62 × 10^−12^	S100A2	2.82 × 10^−3^
COL5A1	1.62 × 10^−12^	POSTN	1.62 × 10^−12^	DNAJB4	1.62 × 10^−12^	S100A8	1.31 × 10^−6^
COL5A2	1.62 × 10^−12^	RAB25	<1 × 10^−12^	EGFR	2.22 × 10^−16^	SAA1	5.55 × 10^−16^
COL6A1	<1 × 10^−12^	S100A14	<1 × 10^−12^	ELK3	1.11 × 10^−16^	SEMA5A	1.62 × 10^−12^
CORO1A	1.62 × 10^−12^	SAMD9	<1 × 10^−12^	FBLN5	1.62 × 10^−12^	SERPINB2	1.03 × 10^−4^
DSP	6.9 × 10^−10^	SERPINE1	8.56 × 10^−11^	FGFBP1	2.13 × 10^−5^	SNAI2	1.74 × 10^−12^
ECM1	1.62 × 10^−12^	SPINT2	1.62 × 10^−12^	GJB3	9.02 × 10^−11^	SOX10	<1 × 10^−12^
EPCAM	1.62 × 10^−12^	SPRR1B	1.26 × 10^−3^	GNG11	<1 × 10^−12^	TBX3	5.36 × 10^−4^
ERBB3	<1 × 10^−12^	ST14	<1 × 10^−12^	IL1B	3.04 × 10^−3^	TFPI	<1 × 10^−12^
ESR1	<1 × 10^−12^	SYK	2.6 × 10^−6^	IL4R	1.00 × 10^−9^	TGFB1I1	<1 × 10^−12^
ESRP1	<1 × 10^−12^	TCF3	1.62 × 10^−12^	ITGA6	<1 × 10^−12^	THBD	1.62 × 10^−12^
ESRP2	1.62 × 10^−12^	TGFB1	1.62 × 10^−12^	ITGB1	1.39 × 10^−13^	TP63	<1 × 10^−12^
FN1	<1 × 10^−12^	THY1	1.62 × 10^−12^	JAG1	3.33 × 10^−16^	TWIST1	7.72 × 10^−12^
FXYD3	1.62 × 10^−12^	TSPAN1	1.62 × 10^−12^	KLF10	<1 × 10^−12^	TWIST2	<1 × 10^−12^
GREM1	2.09 × 10^−9^	VCAN	<1 × 10^−12^	KLK5	2.34 × 10^−8^	VIM	1.62 × 10^−12^
GRHL2	1.62 × 10^−12^			KRT14	1.62 × 10^−12^	WNT5A	1.8 × 10^−2^
ITGA5	2.42 × 10^−4^			KRT15	<1 × 10^−12^	ZEB1	1.62 × 10^−12^
ITGBL1	3.33 × 10^−16^			KRT16	8.79 × 10^−8^	ZEB2	<1 × 10^−12^

**Table 2:** List of genes coherently associated with all survival (DMFS, OS, and RFS). The red and black colors indicate the *p*-value.

**Genes Whose High Expression Correlates with Worse Survival Probability**							
**Gene**	**DMFS**	**OS**	**RFS**	**Gene**	**DMFS**	**OS**	**RFS**
**ABCA12**	3 × 10^−4^	8 × 10^−4^	7.9 × 10^−3^	**KRT16**	9.8 × 10^−9^	1.5 × 10^−5^	9 × 10^−15^
**ADAM9**	1.3 × 10^−7^	3.8 × 10^−5^	4.6 × 10^−13^	**LAD1**	4.4 × 10^−11^	2.4 × 10^−4^	1.4 × 10^−8^
**ARTN**	7.5 × 10^−3^	3.8 × 10^−2^	3.1 × 10^−3^	**LAMA3**	4.9 × 10^−2^	1.8 × 10^−2^	7.1 × 10^−3^
**CD24**	1.4 × 10^−7^	9.2 × 10^−7^	4 × 10^−12^	**LTBP1**	2 × 10^−7^	1.4 × 10^−2^	1.6 × 10^−6^
**CDH2**	1.2 × 10^−4^	8.3 × 10^−6^	4.2 × 10^−7^	**MGAT5B**	1.2 × 10^−2^	1.2 × 10^−2^	2.1 × 10^−5^
**CKMT1A**	2.1 × 10^−16^	3.6 × 10^−8^	7.8 × 10^−14^	**MT2A**	3.8 × 10^−5^	8.2 × 10^−5^	8.5 × 10^−7^
**CKMT1B**	2.1 × 10^−16^	3.6 × 10^−8^	7.8 × 10^−14^	**NDRG1**	4.6 × 10^−11^	3.4 × 10^−7^	< 10^−16^
**CLDN1**	9.7 × 10^−5^	2.1 × 10^−2^	4.5 × 10^−5^	**PLP2**	2.7 × 10^−4^	8 × 10^−3^	3 × 10^−11^
**CLDN3**	2 × 10^−2^	7.4 × 10^−4^	6.4 × 10^−5^	**S100A8**	6.9 × 10^−9^	6.8 × 10^−6^	1.3 × 10^−12^
**CLDN4**	9.5 × 10^−3^	3 × 10^−3^	1.2 × 10^−2^	**SERPINE1**	4.1 × 10^−3^	2.5 × 10^−2^	1.7 × 10^−5^
**CLDN7**	5.3 × 10^−6^	2.1 × 10^−4^	7.3 × 10^−3^	**SLPI**	1.4 × 10^−6^	4.5 × 10^−4^	8.3 × 10^−6^
**EPCAM**	4.5 × 10^−6^	2.1 × 10^−4^	<10^−16^	**SNAI1**	1.8 × 10^−7^	1.6 × 10^−4^	7.4 × 10^−4^
**ESRP1**	3.9 × 10^−7^	3.7 × 10^−5^	8 × 10^−14^	**ST14**	1.6 × 10^−5^	1.5 × 10^−6^	4.6 × 10^−9^
**ESRP2**	5.4 × 10^−4^	4.5 × 10^−5^	3.4 × 10^−4^	**TCF3**	1.5 × 10^−4^	1.8 × 10^−3^	6.6 × 10^−6^
**FN1**	9.7 × 10^−4^	2.9 × 10^−2^	5 × 10^−3^	**TGFB1**	3.6 × 10^−2^	4.6 × 10^−2^	1.6 × 10^−2^
**GREM1**	4.6 × 10^−7^	2.4 × 10^−3^	5.6 × 10^−11^	**THY1**	1.8 × 10^−3^	5.4 × 10^−3^	3.7 × 10^−3^
**ITGB1**	1 × 10^−3^	5.5 × 10^−3^	1.1 × 10^−9^				
**Genes Whose High Expression Correlates with Better Survival Probability**							
**Gene**	**DMFS**	**OS**	**RFS**	**Gene**	**DMFS**	**OS**	**RFS**
**ALDH1A1**	1.1 × 10^−6^	5 × 10^−8^	8.2 × 10^−10^	**KCNMA1**	8.2 × 10^−5^	4.5 × 10^−5^	1.7 × 10^−8^
**AXL**	3.8 × 10^−2^	1.4 × 10^−2^	1.3 × 10^−4^	**KRT14**	1.2 × 10^−4^	2.8 × 10^−3^	6.8 × 10^−7^
**CD44**	3.6 × 10^−8^	7.9 × 10^−5^	3 × 10^−13^	**LIFR**	4.4 × 10^−4^	5.3 × 10^−8^	4.5 × 10^−9^
**CDH1**	3.2 × 10^−2^	4 × 10^−2^	3.5 × 10^−6^	**MLPH**	2.2 × 10^−8^	3.9 × 10^−3^	< 10^−16^
**COL17A1**	2.5 × 10^−3^	7.8 × 10^−4^	8 × 10^−16^	**NR2F1**	2.2 × 10^−2^	1.6 × 10^−2^	4.2 × 10^−2^
**COL6A1**	3.3 × 10^−2^	3.6 × 10^−3^	1.1 × 10^−4^	**PLAT**	5.9 × 10^−10^	2.1 × 10^−4^	1.1 × 10^−11^
**CORO1A**	1.7 × 10^−3^	3.1 × 10^−4^	2.3 × 10^−3^	**PRKCH**	1.4 × 10^−3^	8.3 × 10^−3^	1.6 × 10^−9^
**DCN**	8.7 × 10^−5^	3.4 × 10^−3^	1.7 × 10^−4^	**S100A14**	2.9 × 10^−2^	6 × 10^−3^	6.2 × 10^−3^
**ERBB3**	2.6 × 10^−3^	5.8 × 10^−4^	7.9 × 10^−5^	**SAA1**	1.8 × 10^−5^	4.2 × 10^−3^	6.6 × 10^−5^
**ESR1**	<10^−16^	2.2 × 10^−7^	<10^−16^	**SEMA5A**	1.9 × 10^−2^	1.3 × 10^−2^	5.6 × 10^−6^
**FBLN5**	1.1 × 10^−2^	5.3 × 10^−4^	5.9 × 10^−7^	**SERPINB1**	1.5 × 10^−4^	1.4 × 10^−3^	<10^−16^
**FGFR2**	1.8 × 10^−11^	1.2 × 10^−8^	3.1 × 10^−11^	**TBX3**	1.8 × 10^−4^	5.7 × 10^−4^	3.1 × 10^−10^
**FST**	4.4 × 10^−2^	1.8 × 10^−2^	5 × 10^−7^	**TFPI**	4.3 × 10^−2^	5.5 × 10^−5^	5.4 × 10^−3^
**ITGBL1**	7.6 × 10^−3^	5.2 × 10^−3^	4 × 10^−7^	**TP63**	3.7 × 10^−2^	2.4 × 10^−3^	3.5 × 10^−10^

**Table 3:** Relationship between gene expression of gene involved in EMT in BC and clinical-pathological parameters, including estrogen receptor (ER), progesterone receptor (PR), and human epidermal growth receptor 2 (HER) status, lymph node (N) involvement, and grading (G1-G2-G3). The genes associated with poor and good prognosis, as determined by KM Plotter analysis, are indicated in red and in black, respectively. Data were retrieved for each gene using bcGeneXMiner, and the grading was assessed using the GOBO database. *p* < 0.05 was considered significant (*p* < 0.05 *, *p* < 0.01 **, *p* < 0.001 ***).

**Gene Name**	**ER+/PR+**	**ER−/PR−**	**HER2+**	**HER2−**	**N+**	**N−**	**G1**	**G2**	**G3**
ABCA12	−	−	<0.0001 ***		0.0002 ***			0.00324 **	
ARTN	<0.0001 ***			<0.0001 ***	−	−			0.08766
CD24		<0.0001 ***	<0.0001 ***		0.0009 ***				<0.0001 ***
CDH2		<0.0001 ***	<0.0001 ***		−	−			0.0001 ***
CKMT1A	/	/	<0.0001 ***		−	−	/	/	/
CKMT1B		<0.0001 ***	<0.0001 ***		0.0017 **		/	/	/
CLDN3	/	/	0.0040 **		<0.0001 ***				0.0842
CLDN4		<0.0001 ***	0.0006 ***		0.0002 ***				<0.0001 ***
CLDN7	<0.0001 ***		−	−	<0.0001 ***			<0.0001 ***	
EPCAM		<0.0001 ***	0.0004 ***		0.0073 **				<0.0001 ***
ESRP1		<0.0001 ***	<0.0001 ***		<0.0001 ***				<0.0001 ***
ESRP2	<0.05 *		<0.0001 ***		<0.0001 ***			<0.0001 ***	
FN1	−	−	<0.0001 ***		0.0022 **		0.00321 **		
GREM1		<0.0001 ***	<0.0001 ***		<0.0001 ***			0.02523 *	
LAD1		<0.0001 ***	<0.0001 ***		−	−			<0.0001 ***
MGAT5B		<0.0001 ***	0.0020 **		0.0385 *		/	/	/
PLP2		<0.0001 ***	<0.0001 ***		<0.0001 ***				0.55961
SERPINE1		<0.05 *	−	−	<0.0001 ***			0.00007 ***	
ST14		<0.0001 ***	<0.0001 ***			0.0095 **			0.0002 ***
TCF3		<0.0001 ***	<0.0001 ***		−	−	0.33625		
TGFB1	/	/	−	−	−	−	0.00033 ***		
THY1	−	−	<0.0001 ***		−	−	<0.0001 ***		
**Gene Name**	**ER+/PR+**	**ER−/PR−**	**HER2+**	**HER2−**	**N+**	**N−**	**G1**	**G2**	**G3**
ALDH1A1	<0.01 *		0.0164 *		0.0103 *		0.00292 **		
AXL	<0.0001 ***		−	−	−	−	<0.0001 ***		
CD44		<0.0001 ***		<0.0001 ***		<0.0001 ***	<0.0001 ***		
COL17A1	<0.0001 ***		−	−		< 0.0001 ***	<0.0001 ***		
DCN	<0.0001 ***		−	−	−	−	<0.0001 ***		
FBLN5		<0.0001 ***	−	−	−	−	<0.0001 ***		
KRT14		<0.0001 ***		<0.0001 ***	−	−	0.00002 ***		
LIFR	<0.0001 ***			<0.0001 ***		0.0037 **	0.00836 **		
NR2F1	<0.0001 ***		<0.0001 ***		0.0023 **		<0.0001 ***		
PRKCH		<0.0001 ***	<0.0001 ***		0.0006 ***		<0.0001 ***		
SAA1		<0.0001 ***		<0.0001 ***		0.0015 **	/	/	/
SEMA5A	<0.0001 ***		−	−		0.0372 *	<0.0001 ***		
TBX3	<0.0001 ***			<0.0001 ***		0.0113 *	<0.0001 ***		
TFPI		<0.0001 ***	<0.0001 ***		0.0074 **		<0.0001 ***		
TP63	<0.0001 ***		−	−	−	−	<0.0001 ***		

## Data Availability

All data supporting the findings of this study are provided as Supplementary Materials.

## Supplementary Materials

The following supporting information can be downloaded at: https://www.mdpi.com/article/10.3390/biology15030265/s1, Table S1. Clinicopathological characteristics of breast cancer patients. Table S2. Pearson correlation of EMT markers and clinicopathological characteristics. Table S3. List of 14 selected studies from EMTome used to generate the EMT signature specifically associated with breast cancer. Table S4. List of 144 genes selected by EMTome. Table S5. List of genes and protein abbreviations used in the text. Figure S1. Histogram of α-SMA and E-cadherin expression levels, normalized to β-actin levels, in the cohort of 95 BC tissues analyzed. Patients were sorted by increased levels of Vimentin expression. Figure S2. (a) α-SMA/E-cadherin density ratio on a log_10_ scale. (b) Vimentin/Cytokeratin-18 density ratio on a log_10_ scale. A ratio < 1 (orange) indicates patients with a predominant epithelial phenotype; a ratio between 1 and 10 (yellow) indicates patients with an intermediate phenotype; and a ratio > 10 (green) indicates patients with a predominant mesenchymal phenotype. Patients are ordered according to increasing Vimentin/E-cadherin density ratio. Figure S3. UALCAN analysis of 117 genes found to be significantly different between normal and tumor. Figure S4. Survival analysis (RFS) of the 22 genes, whose expression is associated with a worse prognosis. Genes overexpressed in tumor tissue versus healthy tissue (UALCAN analysis) are identified by upward-facing red arrows. Figure S5. Survival analysis (OS) of the 22 genes, whose expression is associated with a worse prognosis. Genes overexpressed in tumor tissue versus healthy tissue (UALCAN analysis) are identified by upward-facing red arrows. Figure S6. Survival analysis (DMFS) of the 22 genes, whose expression is associated with a worse prognosis. Overexpressed genes in tumor tissue versus healthy tissue (UALCAN analysis) are identified by upward-facing red arrows. Figure S7. Survival analysis (RFS) of the 15 genes, whose expression is associated with a better prognosis. Down-regulated genes in the tumor tissue relative to healthy tissue (UALCAN analysis) are identified by black arrows pointing downwards. Figure S8. Survival analysis (OS) of the 15 genes, whose expression is associated with a better prognosis. Down-regulated genes in tumor tissue versus healthy tissue (UALCAN analysis) are identified by black arrows pointing downwards. Figure S9. Survival analysis (DMFS) of the 15 genes, whose expression is associated with a better prognosis. Down-regulated genes in tumor tissue versus healthy tissue (UALCAN analysis) are identified by black arrows pointing downwards. Figures S10–S19. Original Western blot used.

## Abbreviations

The following abbreviations are used in this manuscript: EMT, Epithelial-to-mesenchymal transition; MET, Mesenchymal-to-epithelial transition; BC, Breast cancer; ECM, Extracellular matrix; EMP, Epithelial–mesenchymal plasticity; CSC, Cancer stem cell; TME, Tumor microenvironment; MW, Molecular weight; KM, Kaplan–Meier; RFS, Relapse free survival; OS, Overall survival; DMFS, Distant metastasis free survival; MMP, Matrix metalloproteinase; ER, Estrogen receptor; IL, Interleukin; PR, Progesterone receptor; HER2, Human epidermal growth factor receptor 2; N, Lymph node status; G1–G3, Tumor grading; TAM, Tumor-associated macrophages; GOBO, Gene expression-based Outcome for Breast Cancer Online; SDS, Sodium dodecyl sulfate; CCLE, Cancer Cell Line Encyclopedia; TCGA, The Cancer Genome Atlas.
