# Supplementary material for: Decoding the Molecular Drivers of Epithelial to Mesenchymal Transition in Breast Cancer: Insights into Epithelial Plasticity and Microenvironment Crosstalk
Source: Biology (Basel). 2026 Feb 1;15(3):265. doi: 10.3390/biology15030265 (PMC12896715; doi:10.3390/biology15030265)
Supplement: Supplementary file 1 [file biology-15-00265-s001.zip › biology-4114618-supplementary.pdf]

Table S1. Clinicopathological characteristics of breast cancer patients.

Patient ID	Age	T	N	Grading	HER2	ER%	PGR%	Ki67%
**1**	67	1	0	2	2	95	10	30
**2**	65	2	0	3	0	95	30	70
**3**	64	2	1	2	1	85	5	17
**4**	63	1	1	1	0	60	70	5
**5**	42	1	1	2	0	40	60	45
**6**	61	1	-	3	0	50	40	-
**7**	63	2	1	3	1	95	90	50
**8**	47	1	0	2	1	80	60	20
**9**	63	2	1	2	0	90	65	14
**10**	67	1	0	3	1	95	20	40
**11**	41	2	1	3	2	80	5	60
**12**	58	2	1	3	0	0	0	85
**13**	56	2	1	3	2	0	0	40
**14**	60	1	0	3	1	0	0	60
**15**	51	1	1	1	1	95	95	7
**16**	58	4	1	3	3	0	0	13
**17**	56	1	0	3	0	0	0	70
**18**	52	2	1	1	1	65	40	12
**19**	43	2	0	2	0	80	30	35
**20**	65	1	1	2	1	95	5	25
**21**	66	2	1	3	3	0	0	35
**22**	64	1	0	2	1	0	0	10
**23**	61	2	0	3	0	3	0	61
**24**	60	2	1	3	3	70	60	20
**25**	53	2	0	3	0	0	0	60
**26**	75	2	1	1	2	95	80	15
**27**	54	1	0	3	2	0	0	85
**28**	44	2	1	3	3	70	60	45
**29**	48	2	0	3	1	85	85	60
**30**	45	2	1	3	0	95	95	60
**31**	75	1	0	3	1	95	40	32
**32**	41	2	0	2	2	75	65	85
**33**	67	2	0	2	1	95	80	20
**34**	49	2	1	3	3	65	85	45
**35**	75	2	1	2	0	95	90	22
**36**	48	1	0	2	2	70	60	18
**37**	37	3	1	3	0	80	0	17
**38**	55	2	1	3	1	90	0	15
**39**	64	2	1	2	1	80	70	9
**40**	57	2	0	2	3	70	70	25
**41**	37	2	1	2	0	95	95	25
**42**	67	2	1	3	0	0	0	25
**43**	64	2	1	3	3	0	5	50

**44**	72	3	1	3	0	0	0	90
**45**	44	2	1	3	0	70	80	40
**46**	31	1	0	3	0	10	0	40
**47**	57	1	0	3	1	0	0	28
**48**	45	2	0	3	0	0	0	60
**49**	67	2	1	3	1	95	95	36
**50**	61	1	1	2	0	95	80	25
**51**	56	2	1	2	2	95	90	15
**52**	64	3	0	3	0	0	0	0
**53**	57	2	1	3	0	95	60	70
**54**	83	3	1	3	-	15	20	-
**55**	65	2	0	1	1	95	95	22
**56**	47	1	0	2	0	70	75	2
**57**	54	2	0	3	0	80	40	-
**58**	47	1	1	2	3	5	5	-
**59**	64	2	1	2	1	85	5	17
**60**	70	2	0	2	-	10	5	-
**61**	72	-	-	3	3	0	0	-
**62**	64	1	0	2	2	5	15	-
**63**	76	2	-	3	0	0	0	-
**64**	51	2	1	3	2	95	75	28
**65**	77	1	-	2	-	5	10	-
**67**		4	0	2	2	5	5	-
**68**	80	2	1	3	0	80	90	-
**69**	73	-	0	-	-	-	-	-
**70**	-	2	1	-	2	0	0	5
**71**	64	2	1	3	3	60	0	-
**73**	51	-	0	3	3	0	0	60
**74**	38	2	0	3	3	0	0	55
**75**	53	1	1	3	0	75	20	30
**76**	60	-	1	3	1	70	80	10
**77**	52	2	1	3	0	5	0	50
**78**	36	2	1	3	3	5	0	42
**79**	46	2	0	3	1	50	70	20
**80**	37	1	1	2	2	70	70	25
**81**	59	2	1	3	0	85	25	40
**82**	60	2	0	2	0	85	5	20
**83**	52	3	0	3	1	80	40	60
**84**	75	4	1	3	0	60	0	5
**87**	66	2	0	2	1	90	80	11
**88**	78	3	1	3	1	0	0	28
**89**	56	1	0	3	3	75	5	40
**92**	44	1	1	3	3	30	10	45
**94**	56	1	1	3	3	80	5	30
**95**	66	2	1	2	1	90	85	20

Table S2. Pearson correlation of EMT markers and clinicopathological characteristics.

Pearson correlation	**Age**	**E-Cadherin**	**Vimentin**	**Cytokerarin-18**	**α-SMA**	**Grading**	**HER2**	**ER%**	**PGR%**	**Ki67%**
**Age**		-	-	-	-	-	-	-	-	-
**E-Cadherin**	-		r = 0,378<br>p =0,00016	r = 0,208<br>p =0,045	r = 0,332<br>p =0,0011	-	-	-	-	-
**Vimentin**	-	r = 0,378<br>p =0,00016		r = 0,609<br>p= 1,017E-10	-	-	-	-	-	r = -0,326<br>p = 0,0043
**Cytokeratin-18**	-	r = 0,208<br>p =0,045	r = 0,609<br>p= 1,017E-10		-	-	-	-	-	r = -0,246<br>p = 0,035
**α-SMA**	-	r = 0,332<br>p =0,0011	-	-		-	-	-	-	-
**Grading**	-	-	-	-	-		-	r= -0,328<br>p = 0,002	r= -0,391<br>p = 0,00020	r= 0,503<br>p = 5,011E-10
**HER2**	-	-	-	-	-	-		-	-	-
**ER%**	-	-	-	-	-	r= -0,328<br>p = 0,002	-		r=0,6887408<br>p = 1,66E-13	r = -0,308<br>p = 0,0071
**PGR%**	-	-	-	-	-	r= -0,391<br>p = 0,00020	-	r=0,6887408<br>p = 1,66E-13		r = -0,242<br>p = 0,037
**Ki67%**	-	-	r = -0,326<br>p = 0,0043	r = -0,246<br>p = 0,035	-	r= 0,503<br>p = 5,011E-10	-	r = -0,308<br>p = 0,0071	r = -0,242<br>p = 0,037	

Table S3. List of 14 selected studies from EMTome used to generate the EMT signature specifically associated with breast cancer.

	Signature	Pubmed Identification	Description	Source	Cancer type	Genes / markers
1	Alsuliman et al.2015	PMID: 26245467	17 EMT signature genes	Microarray	Breast Cancer	17
2	Berardi et al.2015	PMID: 26044847	ATRA treatment modulated EMT signature	qPCR	Breast cancer	27
3	Cheng et al.2014	PMID: 25060555	A signature of EM plasticity and stromal activation in primary tumor	Microarray	Breast Cancer	51
4	Choi et al.2010	PMID: 20215510	A cell line-derived EMT signature	Microarray	Breast Cancer	200
5	Creighton et al.2009	PMID: 19666588	EMT profile in CD44(+)/CD24(-/low)-MS and claudin-low signatures	Microarray	Breast Cancer	15
6	Cursons et al.2015	PMID: 25975820	EGF- and/or hypoxia-induced EMT (MDA-MB-468, PMC42-ET, PMC42-LA)	RNAseq	Breast cancer	206
7	Grosse-Wilde et al.2015	PMID: 26020648	EM expression signature and stemness	Microarray	Breast Cancer	60
8	Iseri et al.2011	PMID: 21177063	Multidrug resistant MCF-7 associated EMT	Microarray	Breast Cancer	15
9	Javaid et al.2015	PMID: 25678598	26-gene EMT RNA signature	shRNA library screen	Breast Cancer	26
10	Jordan et al.2013	PMID: 23716599	EMT gene signature shared between epithelial stem cells developmentally entering EMT and Claudin-low breast cancer cells	Microarray	Breast Cancer	137
11	Remšík et al.2018	PMID: 29462126	EMT associated 10-molecule surface signature	Antibody-based cell surface screening and flow cytometry	Breast Cancer	10
12	Soundararajan et al.2015	PMID: 26123483	EMT-/stem-cell markers	DMFS- and RFS analyses	Breast Cancer	39
13	Taube et al.2010	PMID: 20713713	Core EMT signature	Microarray	Breast Cancer	247
14	Thompson et al.2020	PMID: 31683225	EMT signature to immune checkpoint blockade in lung cancer	Literature	Breast Cancer	12

Table S4. List of 144 genes selected by EMTome.

Number	Gene Symbol*	Gene name	Derived from EMTOME Signature**
1	ABCA12	ATP binding cassette subfamily A member 12	4-13
2	ABCC4	ATP binding cassette subfamily C member 4	4-10
3	ABLIM1	Actin binding LIM protein 1	6-13
4	ACKR3	Atypical chemokine receptor 3	3-10
5	ACSL4	Acyl-CoA synthetase long chain family member 4	4-10
6	ADAM9	ADAM metallopeptidase domain 9	3-6
7	ALDH1A1	Aldehyde dehydrogenase 1 family member A1	6-12
8	ALDH1A3	Aldehyde dehydrogenase 1 family member A3	7-13
9	ANXA8	Annexin A8	7-13
10	ARTN	Artemin	6-13
11	AXL	AXL receptor tyrosine kinase	6-10
12	C1orf116	Chromosome 1 open reading frame 116	6-13
13	CCN2	Cellular communication network factor 2	9-13
14	CD24	CD24 molecule	4-7-11
15	CD44	CD44 molecule (Indian blood group)	11-12
16	CDH1	Cadherin 1	1-4-5-7-8-9-12-13-14
17	CDH2	Cadherin 2	5-8-9-10-12-13
18	CDH3	Cadherin 3	13-14
19	CHN1	Chimerin 1	4-13
20	CKMT1A	Creatine kinase, mitochondrial 1A	4-13
21	CKMT1B	Creatine kinase, mitochondrial 1B	4-7-13
22	CLDN1	Claudin 1	8-13
23	CLDN3	Claudin 3	1-4-8
24	CLDN4	Claudin 4	1-4-8-9-14
25	CLDN7	Claudin 7	1-4-8
26	COL17A1	Collagen type XVII alpha 1 chain	6-7-13
27	COL1A1	Collagen type I alpha 1 chain	3-7
28	COL1A2	Collagen type I alpha 2 chain	2-3-13
29	COL3A1	Collagen type III alpha 1 chain	3-13
30	COL5A1	Collagen type V alpha 1 chain	3-10
31	COL5A2	Collagen type V alpha 2 chain	7-13
32	COL6A1	Collagen type VI alpha 1 chain	7-10
33	CORO1A	Coronin 1A	10-13
34	DCBLD2	Discoidin, CUB and LCCL domain containing 2	4-6
35	DCN	Decorin	7-13
36	DNAJB4	DnaJ heat shock protein family (Hsp40) member B4	4-13
37	DSP	Desmoplakin	1-5
38	ECM1	Extracellular matrix protein 1	6-10-13
39	EGFR	Epidermal growth factor receptor	2-8-10
40	ELK3	ETS transcription factor ELK3	4-6-10
41	EPCAM	Epithelial cell adhesion molecule	4-7-13-14
42	ERBB3	Erb-b2 receptor tyrosine kinase 3	4-12

43	ESR1	Estrogen receptor 1	8-12
44	ESRP1	Epithelial splicing regulatory protein 1	4-13
45	ESRP2	Epithelial splicing regulatory protein 2	4-13
46	FBLN5	Fibulin 5	7-13
47	FGFBP1	Fibroblast growth factor binding protein 1	7-13
48	FGFR2	Fibroblast growth factor receptor 2	6-13
49	FN1	Fibronectin 1	1-2-5-12
50	FOXC2	Forkhead box C2	2-5-12
51	FST	Follistatin	7-13
52	FXYD3	FXYD domain containing ion transport regulator 3	4-7
53	GJB3	Gap junction protein beta 3	6-9-13
54	GNG11	G protein subunit gamma 11	7-10
55	GREM1	Gremlin 1, DAN family BMP antagonist	7-13
56	GRHL2	Grainyhead like transcription factor 2	4-13
57	GSC	Goosecoid homeobox	5-12
58	IL1B	Interleukin 1 beta	7-13
59	IL4R	Interleukin 4 receptor	6-13
60	ITGA5	Integrin subunit alpha 5	2-3
61	ITGA6	Integrin subunit alpha 6	6-11
62	ITGB1	Integrin subunit beta 1	2-11
63	ITGBL1	Integrin subunit beta like 1	6-7
64	JAG1	Jagged canonical Notch ligand 1	2-3
65	KCNMA1	Potassium calcium-activated channel subfamily M alpha 1	6-7
66	KLF10	Kruppel like factor 10	6-12
67	KLK10	Kallikrein related peptidase 10	7-13
68	KLK5	Kallikrein related peptidase 5	7-13
69	KLK8	Kallikrein related peptidase 8	7-13
70	KRT14	Keratin 14	7-13
71	KRT15	Keratin 15	6-13
72	KRT16	Keratin 16	6-13
73	KRT17	Keratin 17	7-13
74	KRT18	Keratin 18	9-12-13
75	KRT5	Keratin 5	7-12
76	KRT6B	Keratin 6B	7-13
77	LAD1	Ladinin 1	4-7-13
78	LAMA3	Laminin subunit alpha 3	6-7
79	LIFR	LIF receptor subunit alpha	4-10
80	LRRC1	Leucine rich repeat containing 1	4-13
81	LTBP1	Latent transforming growth factor beta binding protein 1	7-13
82	MAL2	Mal, T cell differentiation protein 2 (gene/pseudogene)	4-14
83	MCAM	Melanoma cell adhesion molecule	4-10
84	MGAT5B	Alpha-1,6-mannosylglycoprotein 6-beta-N-acetylglucosaminyltransferase B	4-6
85	MLPH	Melanophilin	4-13
86	MMP2	Matrix metallopeptidase 2	1-2-5-13-14
87	MMP3	Matrix metallopeptidase 3	1-2-5
88	MMP9	Matrix metallopeptidase 9	2-5-10

89	MST1R	Macrophage stimulating 1 receptor	2-13
90	MT2A	Metallothionein 2A	6-10
91	NDRG1	N-myc downstream regulated 1	10-13
92	NECTIN3	Nectin cell adhesion molecule 3	10-13
93	NR2F1	Nuclear receptor subfamily 2 group F member 1	7-13
94	NREP	Neuronal regeneration related protein	7-13
95	NRP1	Neuropilin 1	6-13
96	OCLN	Occludin	4-8-9
97	PCOLCE	Procollagen C-endopeptidase enhancer	7-13
98	PKP3	Plakophilin 3	4-9
99	PLAT	Plasminogen activator, tissue type	6-10
100	PLIN3	Perilipin 3	3-6
101	PLP2	Proteolipid protein 2	6-10
102	PLPP3	Phospholipid phosphatase 3	10-13
103	PMP22	Peripheral myelin protein 22	10-13
104	POSTN	Periostin	3-13
105	PRKCH	Protein kinase C eta	4-13
106	PRRX1	Paired related homeobox 1	12-13
107	PTX3	Pentraxin 3	7-13
108	RAB25	RAB25, member RAS oncogene family	4-13
109	RGL1	Ral guanine nucleotide dissociation stimulator like 1	10-13
110	S100A14	S100 calcium binding protein A14	4-7-13
111	S100A2	S100 calcium binding protein A2	6-9
112	S100A8	S100 calcium binding protein A8	7-13
113	SAA1	Serum amyloid A1	7-13
114	SAMD9	Sterile alpha motif domain containing 9	4-6
115	SEMA5A	Semaphorin 5A	3-13
116	SERPINB1	Serpin family B member 1	6-10-13
117	SERPINB2	Serpin family B member 2	6-13
118	SERPINE1	Serpin family E member 1	6-9
119	SLPI	Secretory leukocyte peptidase inhibitor	7-13
120	SNAI1	Snail family transcriptional repressor 1	1-5-9-12
121	SNAI2	Snail family transcriptional repressor 2	1-3-5-8-9-12-14
122	SOX10	SRY-box transcription factor 10	1-5
123	SPINT2	Serine peptidase inhibitor, Kunitz type 2	4-7-13
124	SPRR1B	Small proline rich protein 1B	7-13
125	ST14	Suppression of tumorigenicity 14	4-13-14
126	SYK	Spleen associated tyrosine kinase	10-13
127	TBX3	T-box transcription factor 3	10-13
128	TCF3	Transcription factor 3	9-12
129	TFPI	Tissue factor pathway inhibitor	7-10
130	TGFB1	Transforming growth factor beta 1	2-12
131	TGFB1I1	Transforming growth factor beta 1 induced transcript 1	3-13
132	THBD	Thrombomodulin	10-13
133	THY1	Thy-1 cell surface antigen	3-10-12-13
134	TNC	Tenascin C	3-6
135	TP63	Tumor protein p63	7-13

136	TSPAN1	Tetraspanin 1	4-6-13
137	TWIST1	Twist family bHLH transcription factor 1	1-2-3-5-9-12
138	TWIST2	Twist family bHLH transcription factor 2	1-12
139	VCAN	Versican	2-3
140	VIM	Vimentin	1-2-5-6-8-9-10-12-13-14
141	WNT5A	Wnt family member 5A	2-7-13
142	XDH	Xanthine dehydrogenase	6-13
143	ZEB1	Zinc finger E-box binding homeobox 1	1-9-12-13
144	ZEB2	Zinc finger E-box binding homeobox 2	1-9-12-14

* Iperlink to https://www.genecards.org/

**Signature number from Table S1

Table S5. List of genes and protein abbreviations used in the text.

Gene/protein acronym	Gene/protein name
ABCA12	ATP Binding Cassette subfamily A member 12
ALDH1A1	Aldehyde Dehydrogenase 1 family, member A1
α-SMA	Alpha-Smooth Muscle Actin
AXL	AXL Receptor Tyrosine Kinase
CD24	Cluster Differentiation 24
CD44	Cluster Differentiation 44
CDH1	Cadherin-1 (E-cadherin)
CDH2	Cadherin-2 (N-cadherin)
COL17A1	Collagen type XVII Alpha 1 chain
DCN	Decorin
EGF	Epidermal Growth Factor
EPCAM	Epithelial Cell Adhesion Molecule
ERK	Extracellular Signal-Regulated Kinases
ESRP1	Epithelial Splicing Regulatory Protein 1
ESRP2	Epithelial Splicing Regulatory Protein 2
FBLN5	Fibulin 5
FN1	Fibronectin 1
GREM1	Gremlin 1, DAN Family BMP Antagonist
KRT8	Cytokeratin-8
KRT18	Cytokeratin-18
LAD1	Ladinin 1
LIF	Leukemia Inhibitory Factor
LIFR	Leukemia Inhibitory Factor Receptor Subunit Alpha
MGAT5	Alpha-1,6-Mannosylglycoprotein 6-Beta-N-Acetylglucosaminyltransferase
PLP2	Proteolipid Protein 2
PRKCH	Protein Kinase C Eta
SAA1	Serum Amyloid A1
SDC2	Syndecan 2

SERPINE1	Serpin Family E Member 1
SPRR1B	Small Proline Rich Protein 1B
ST14	ST14 Transmembrane Serine Protease Matriptase
TCF3/TCF7L1	Transcription Factor 3
TGF-β	Transforming Growth Factor Beta 1
Thy-1/CD90	Thy-1 Cell Surface Antigen
VEGFR-2	Vascular Endothelial Growth Factor Receptor 2
VIM	Vimentin
