# Supplementary material for: Decoding the Molecular Drivers of Epithelial to Mesenchymal Transition in Breast Cancer: Insights into Epithelial Plasticity and Microenvironment Crosstalk
Source: Biology (Basel). 2026 Feb 1;15(3):265. doi: 10.3390/biology15030265 (PMC12896715; doi:10.3390/biology15030265)
Supplement: Supplementary file 1 [file biology-15-00265-s001.zip › biology-4114618-supplementary.pptx]

## Slide 1
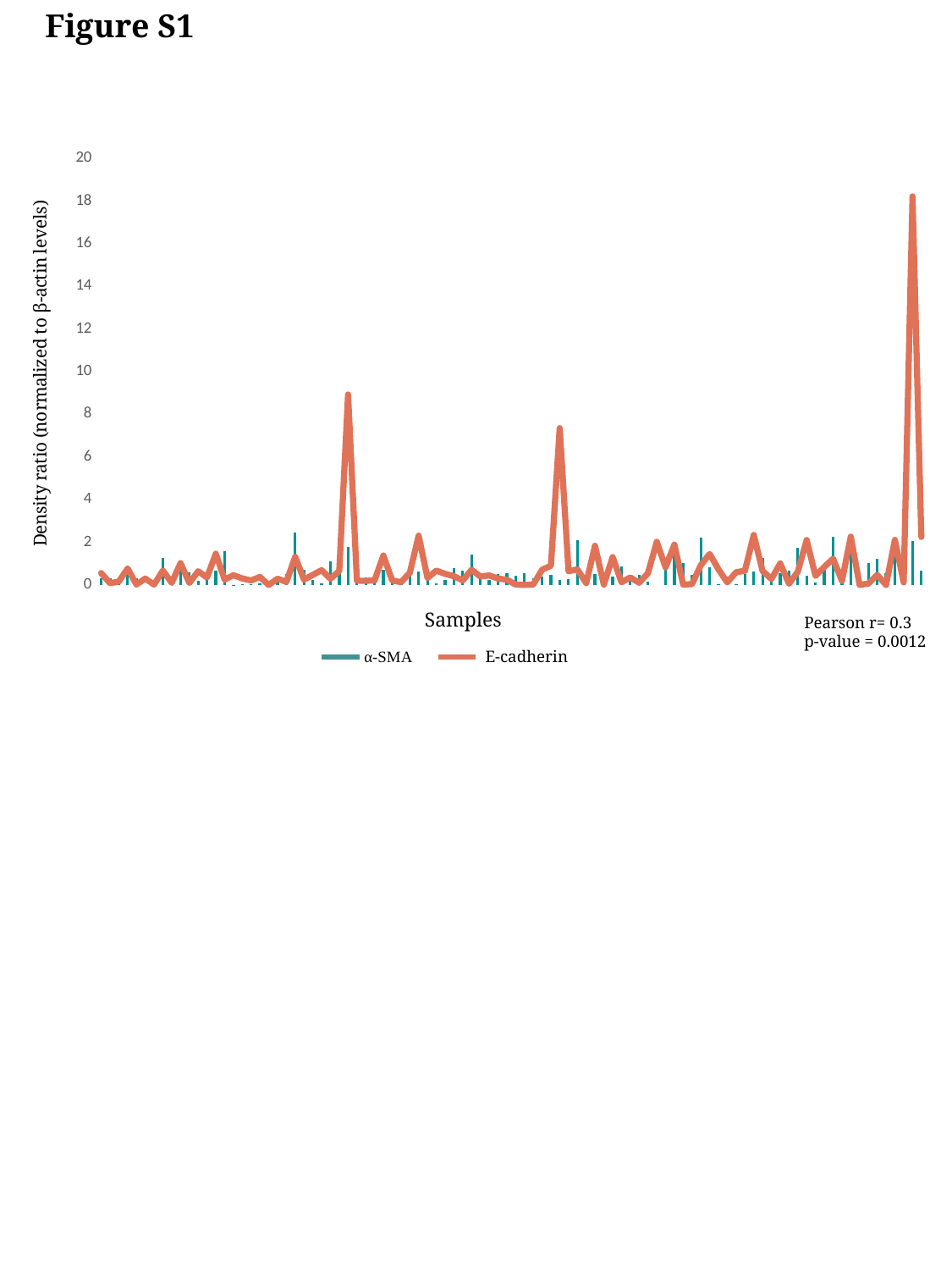

Figure S1
### Chart
| Category | | |
|---|---|---|Samples
Pearson r= 0.3
p-value = 0.0012
α-SMA
E-cadherin

## Slide 2
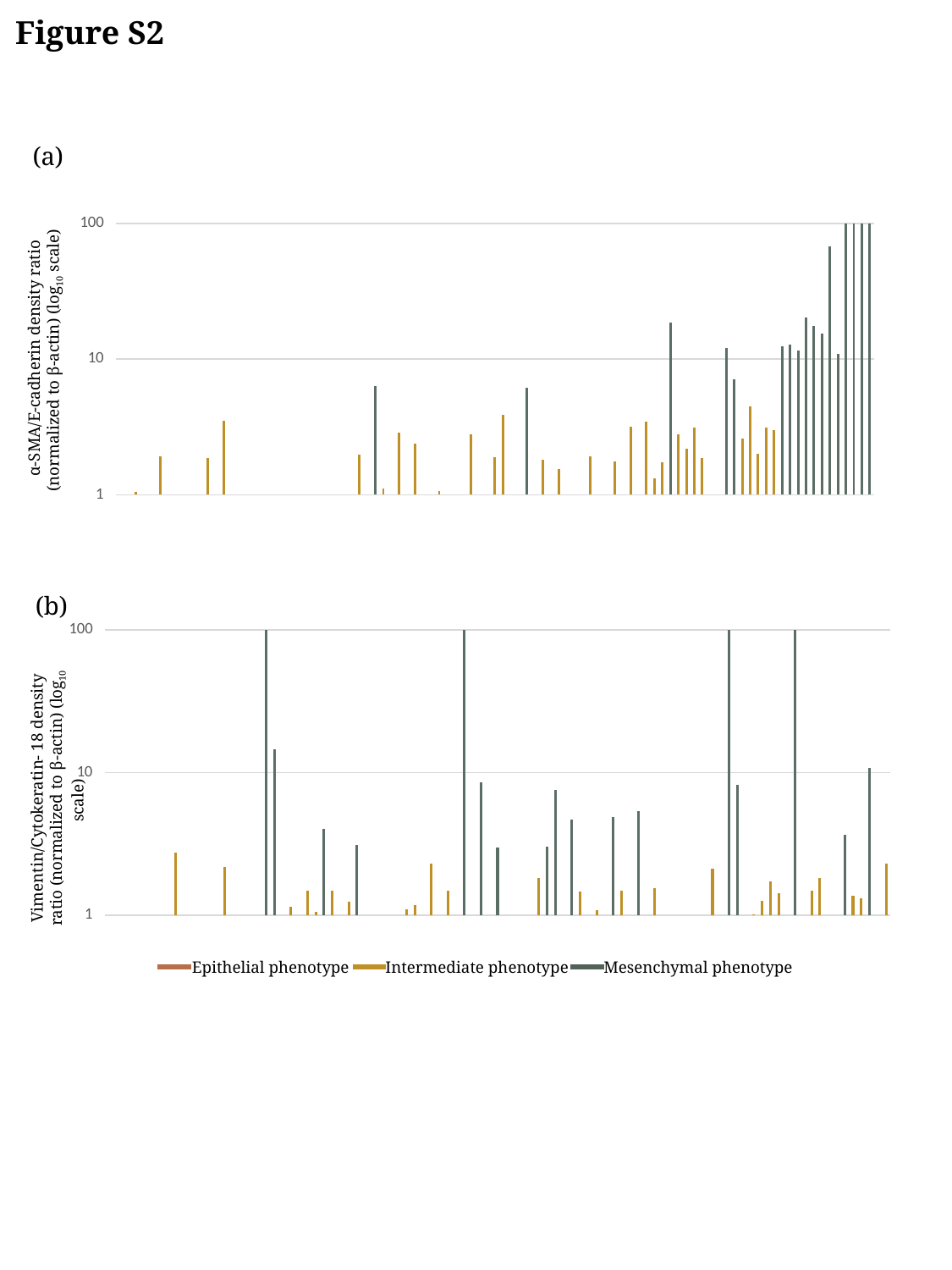

Figure S2
(a)
### Chart
| Category | |
|---|---|α-SMA/E-cadherin density ratio (normalized to β-actin) (log10 scale)
(b)
### Chart
| Category | |
|---|---|Vimentin/Cytokeratin- 18 density ratio (normalized to β-actin) (log10 scale)
Epithelial phenotype
Intermediate phenotype
Mesenchymal phenotype

## Slide 3
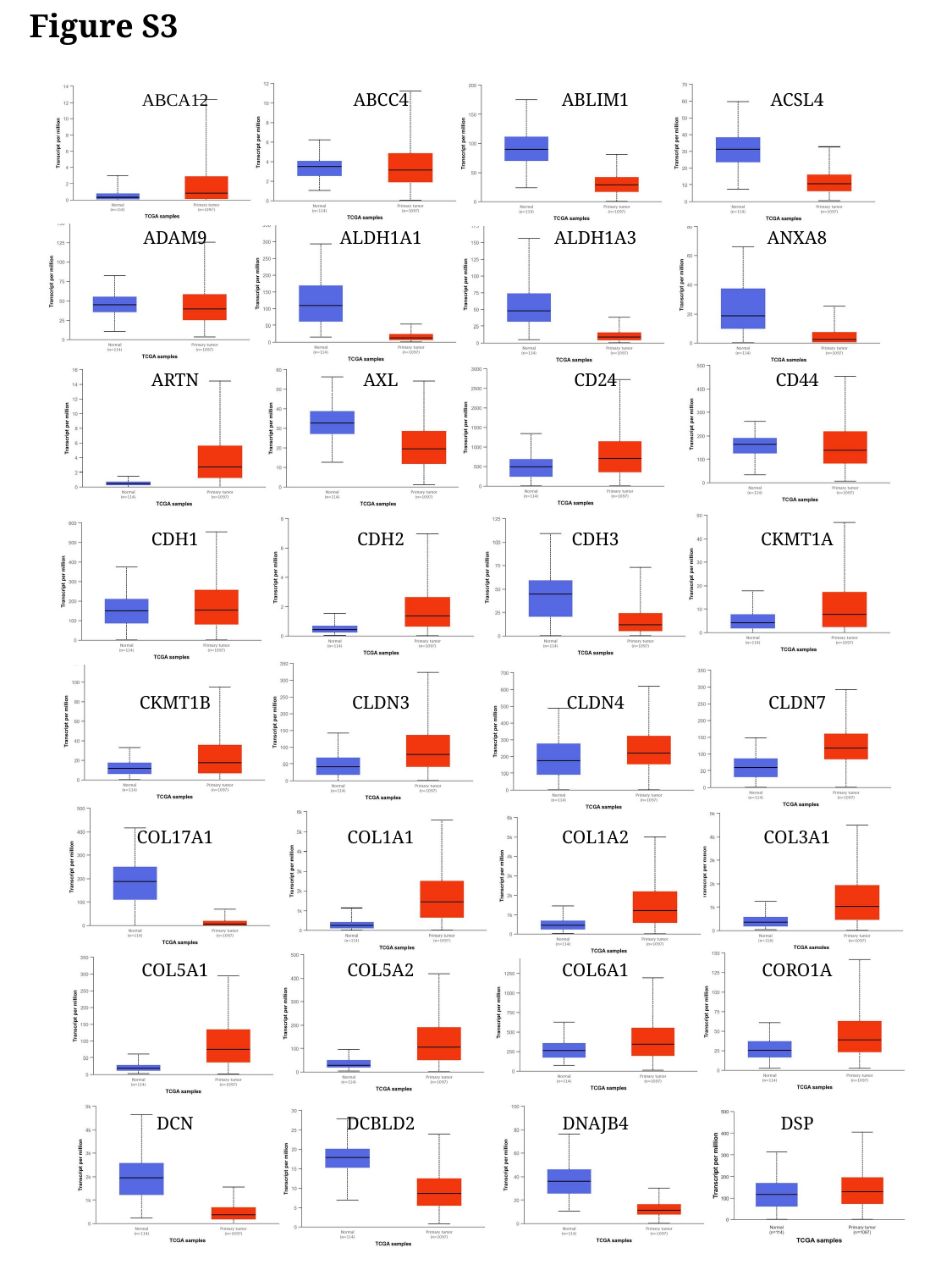

Figure S3
ABCA12
ABCC4
ABLIM1
ACSL4
ANXA8
ADAM9
ALDH1A1
ALDH1A3
ARTN
AXL
CD24
CD44
CDH1
CDH2
CDH3
CKMT1A
CKMT1B
CLDN3
CLDN4
CLDN7
COL17A1
COL1A1
COL1A2
COL3A1
COL5A1
COL5A2
COL6A1
CORO1A
DCN
DCBLD2
DNAJB4
DSP

## Slide 4
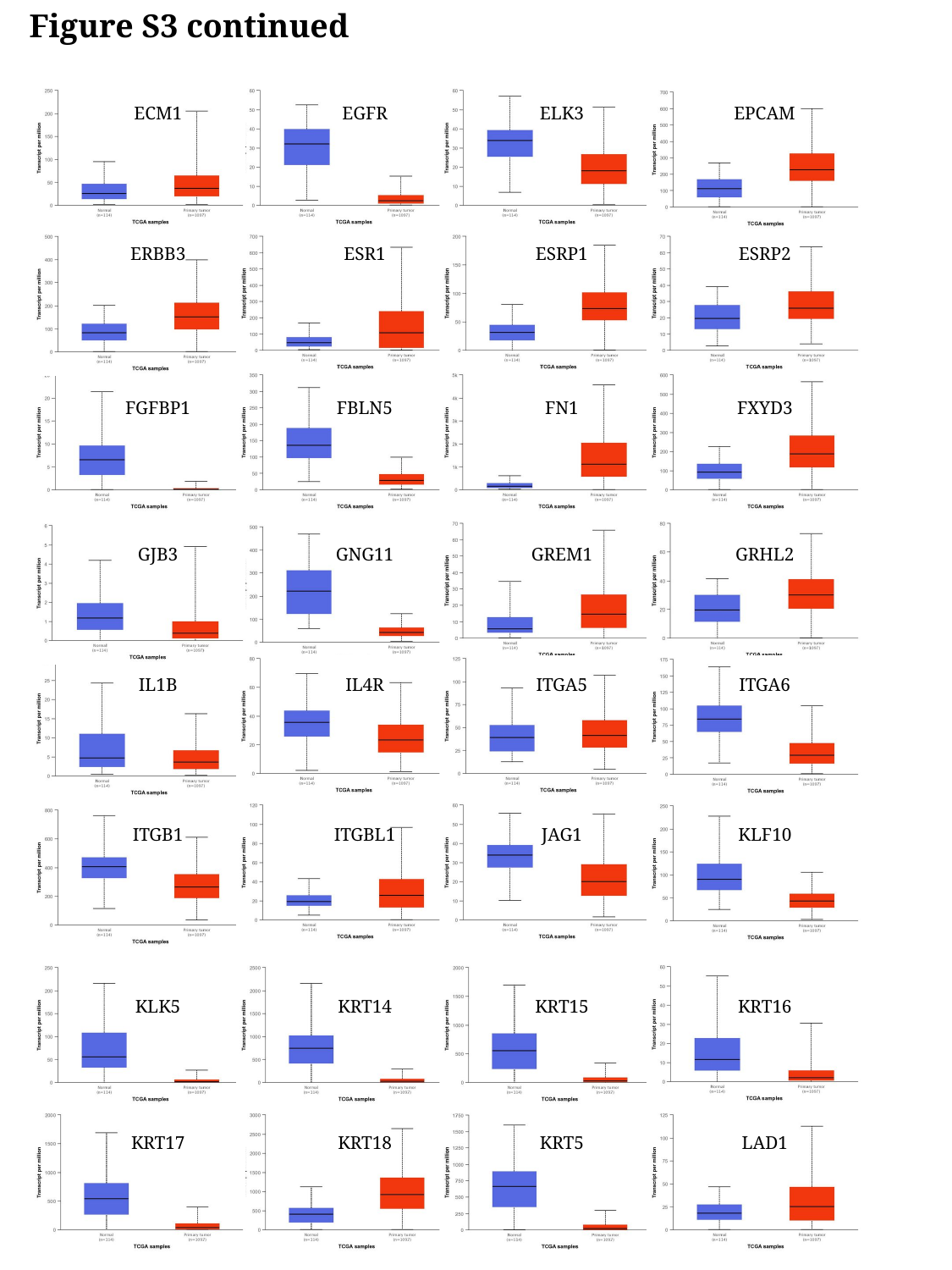

Figure S3 continued
ECM1
EGFR
ELK3
EPCAM
ERBB3
ESR1
ESRP1
ESRP2
FGFBP1
FBLN5
FN1
FXYD3
GJB3
GNG11
GREM1
GRHL2
IL1B
IL4R
ITGA5
ITGA6
ITGB1
ITGBL1
JAG1
KLF10
KLK5
KRT14
KRT15
KRT16
KRT18
KRT5
LAD1
KRT17

## Slide 5
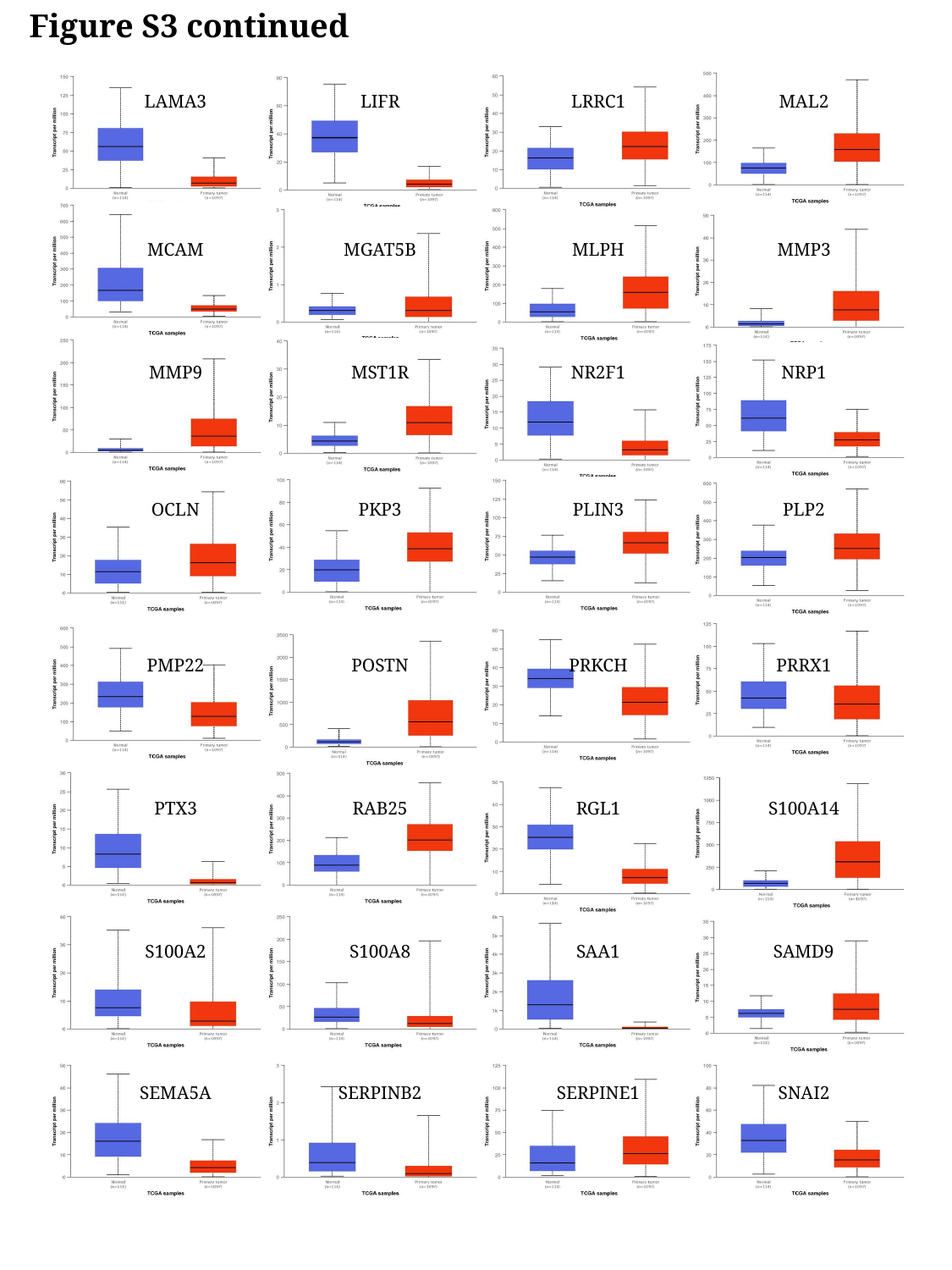

Figure S3 continued
LAMA3
LIFR
LRRC1
MAL2
MMP3
MCAM
MGAT5B
MLPH
MMP9
MST1R
NR2F1
NRP1
OCLN
PKP3
PLIN3
PLP2
PMP22
POSTN
PRKCH
PRRX1
RGL1
S100A14
PTX3
RAB25
S100A2
S100A8
SAA1
SAMD9
SEMA5A
SERPINB2
SERPINE1
SNAI2

## Slide 6
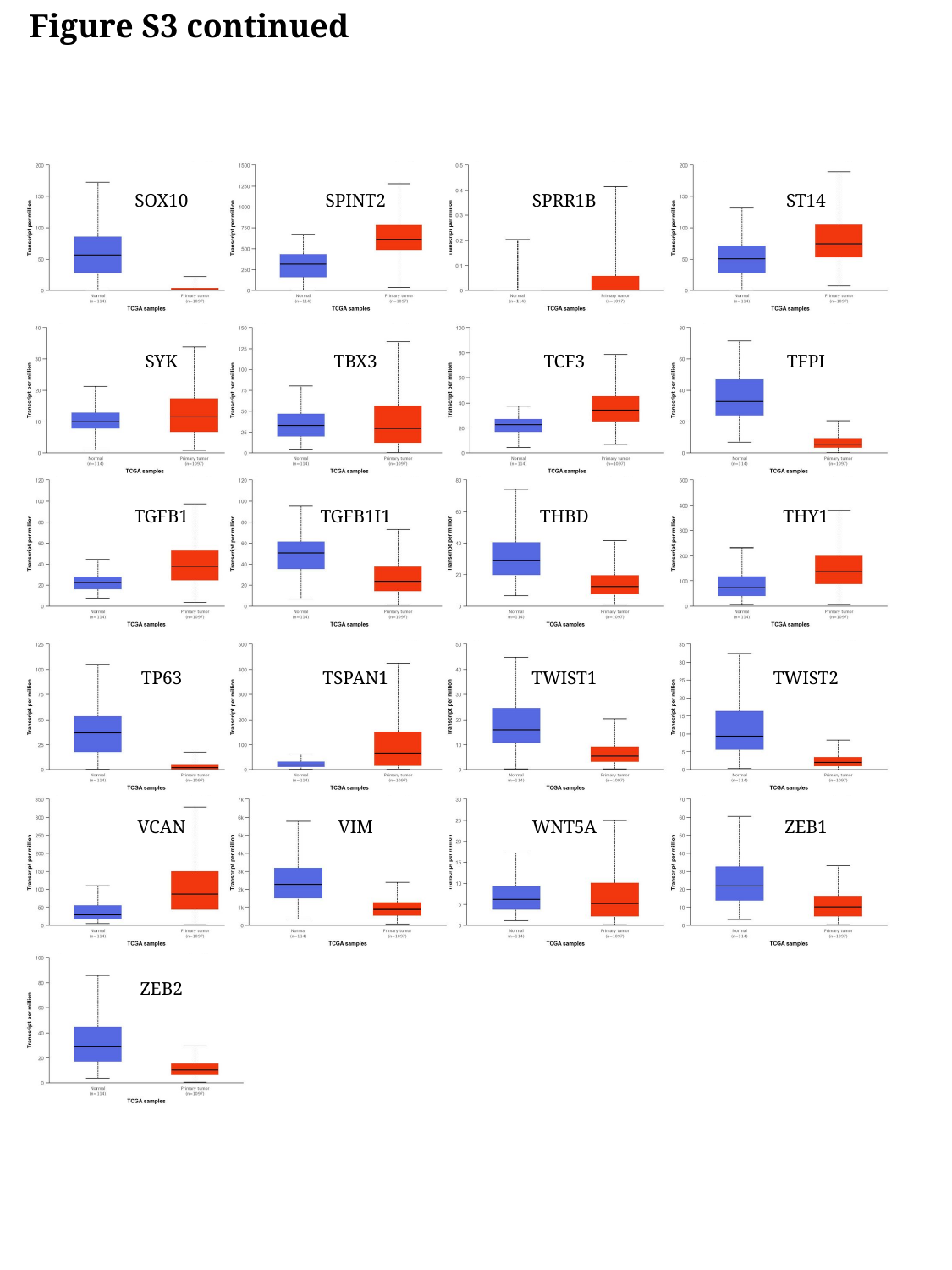

Figure S3 continued
SOX10
SPINT2
SPRR1B
ST14
SYK
TBX3
TCF3
TFPI
TGFB1
TGFB1I1
THBD
THY1
TP63
TSPAN1
TWIST1
TWIST2
VCAN
VIM
WNT5A
ZEB1
ZEB2

## Slide 7
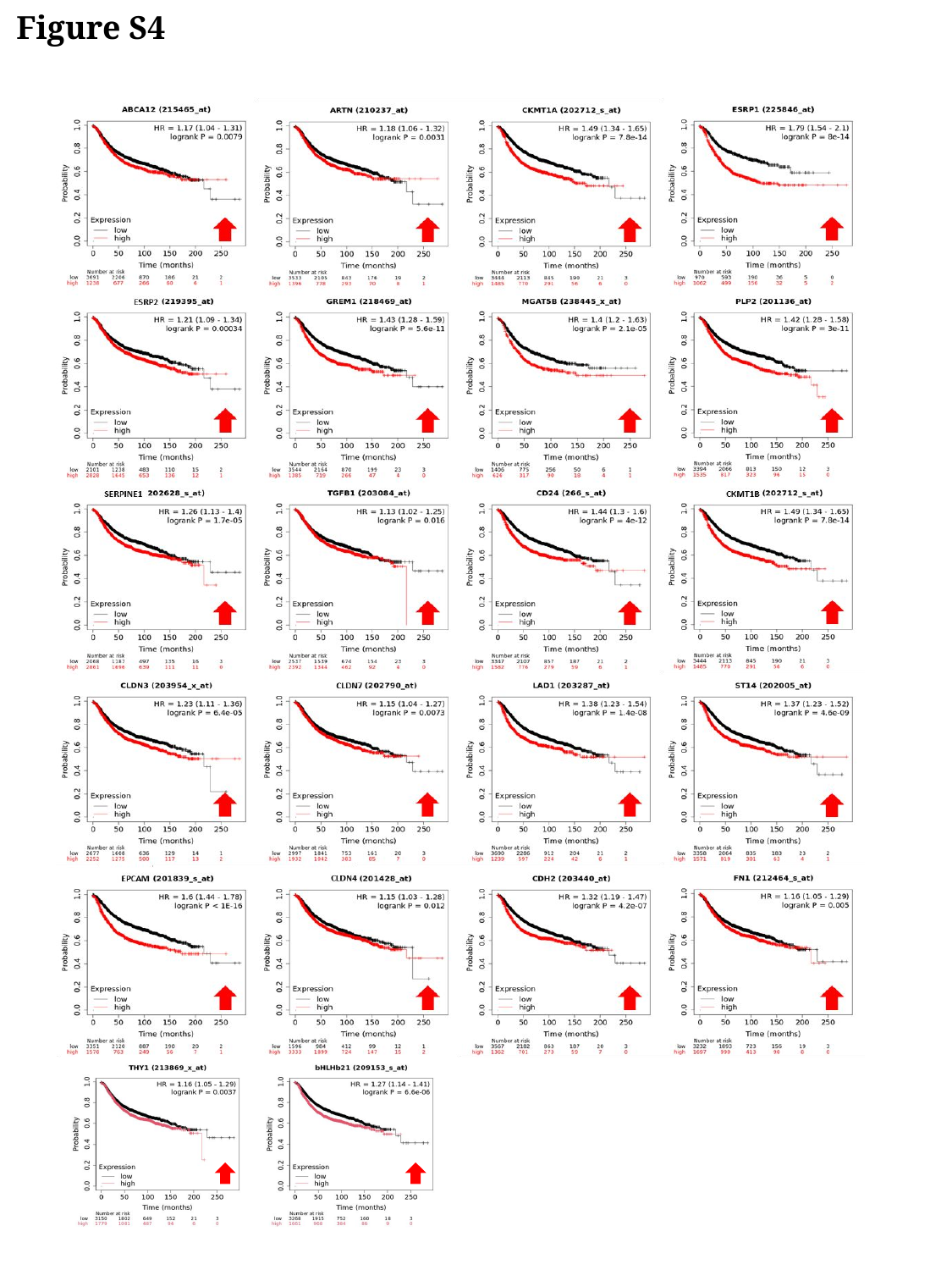

Figure S4

## Slide 8
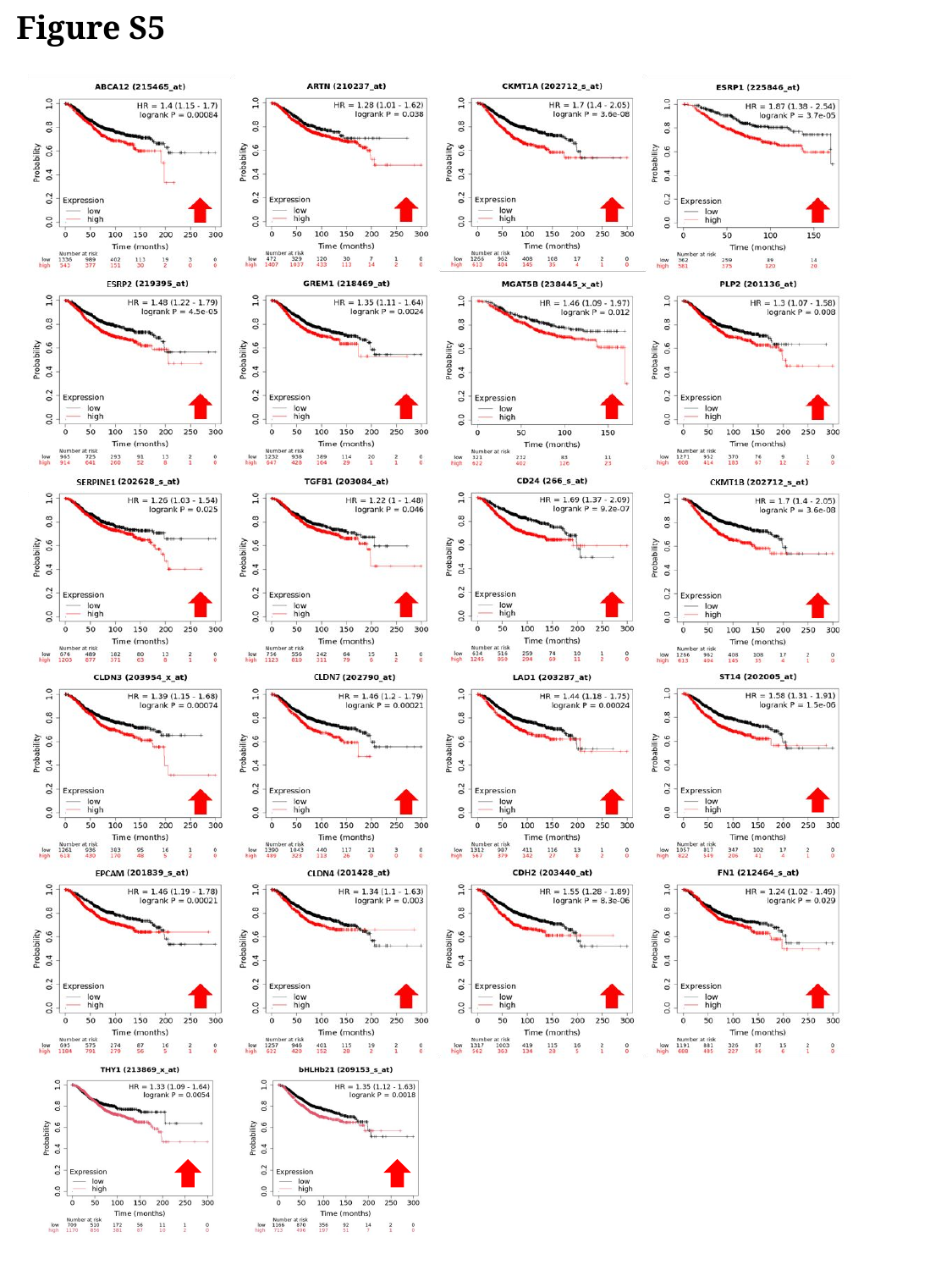

Figure S5

## Slide 9
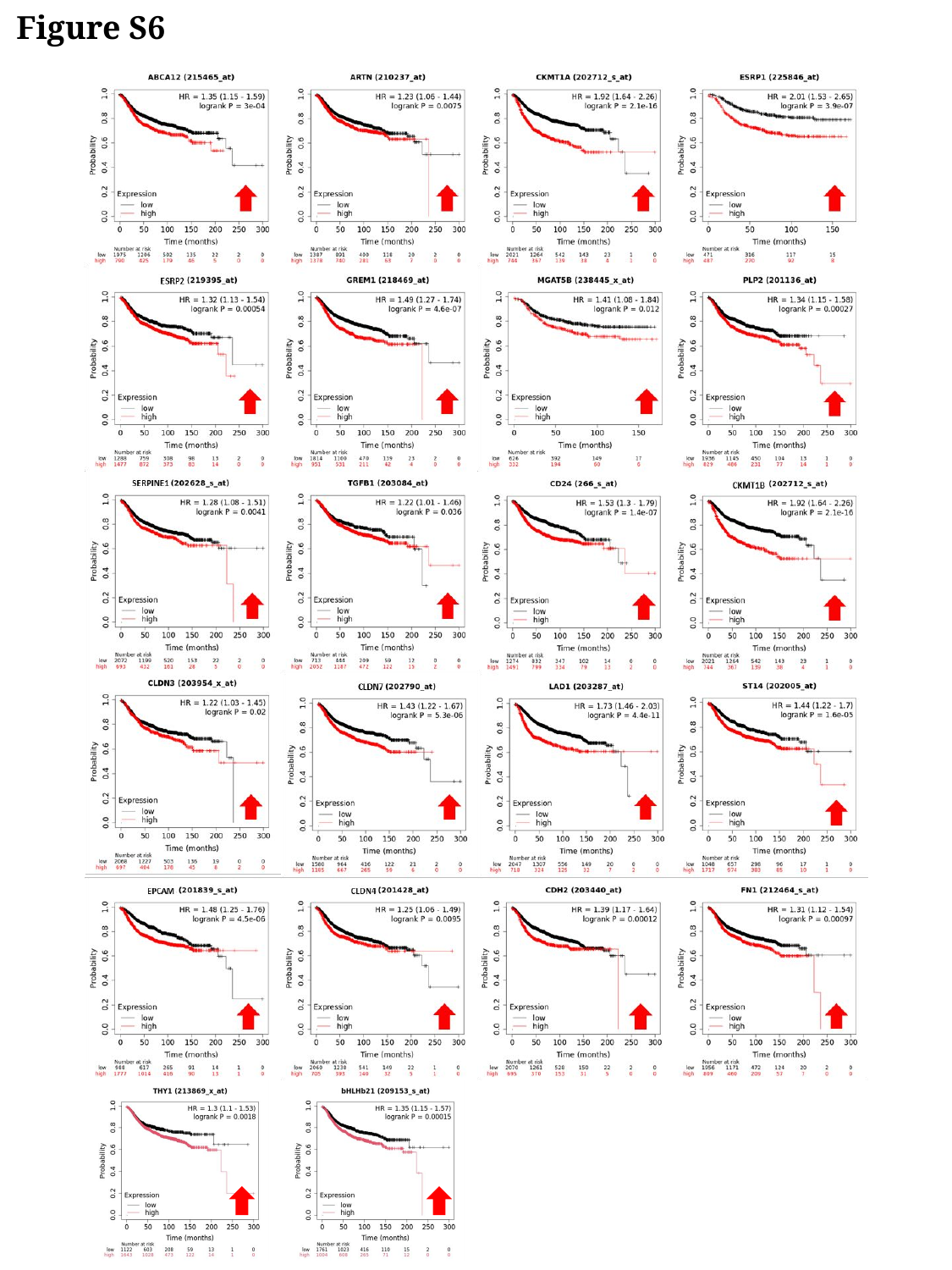

Figure S6

## Slide 10
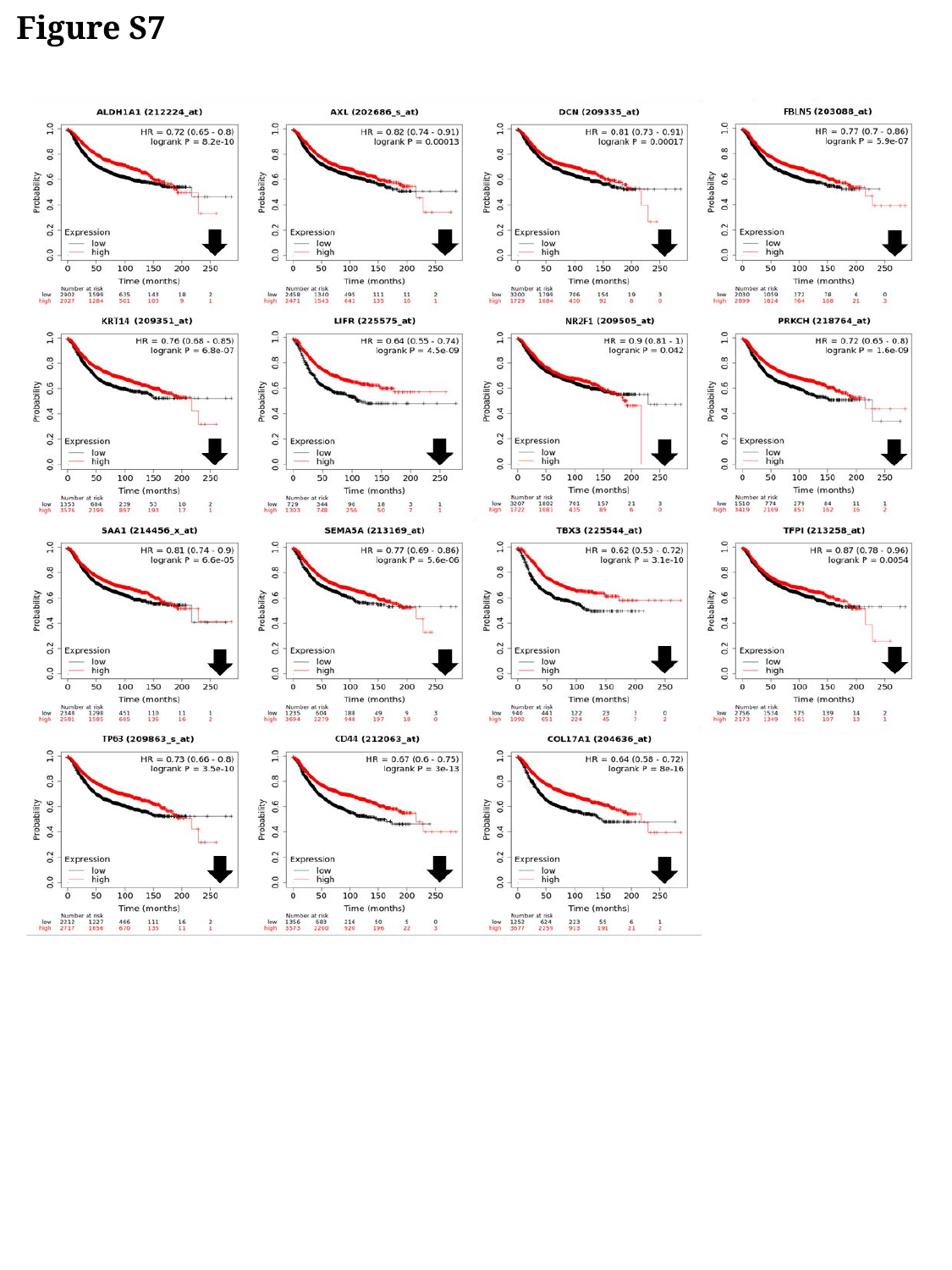

Figure S7

## Slide 11
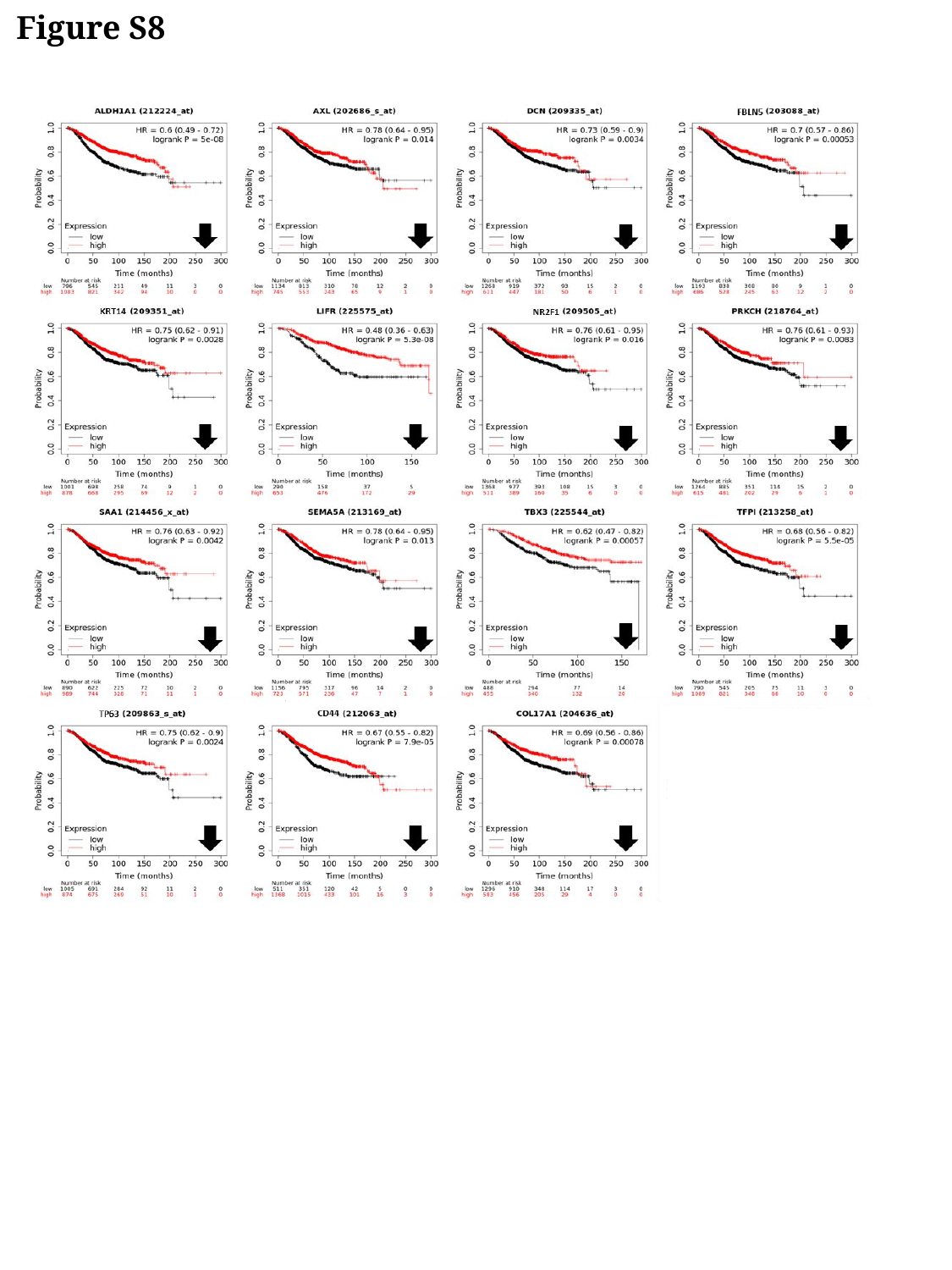

Figure S8

## Slide 12
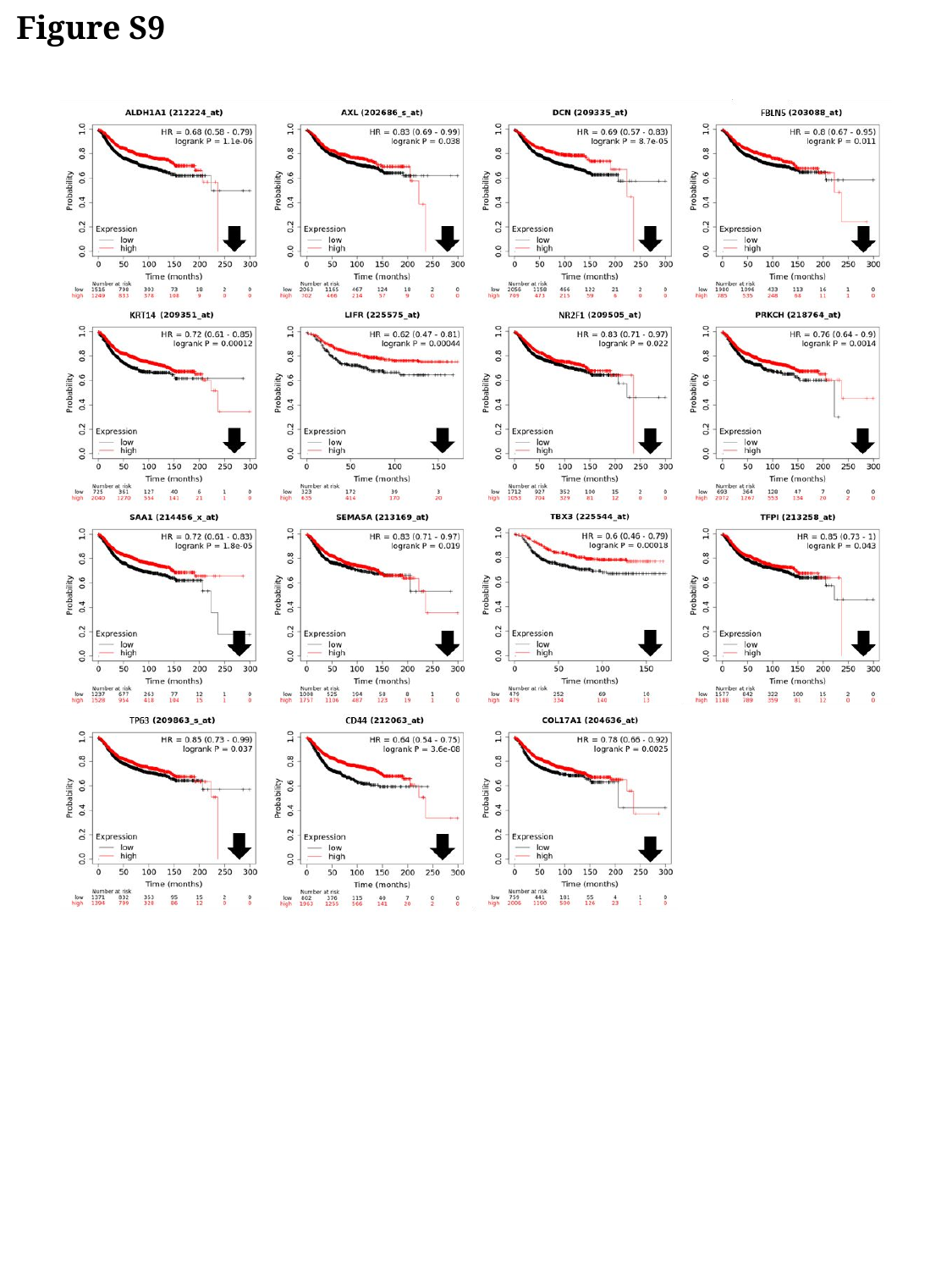

Figure S9

## Slide 13
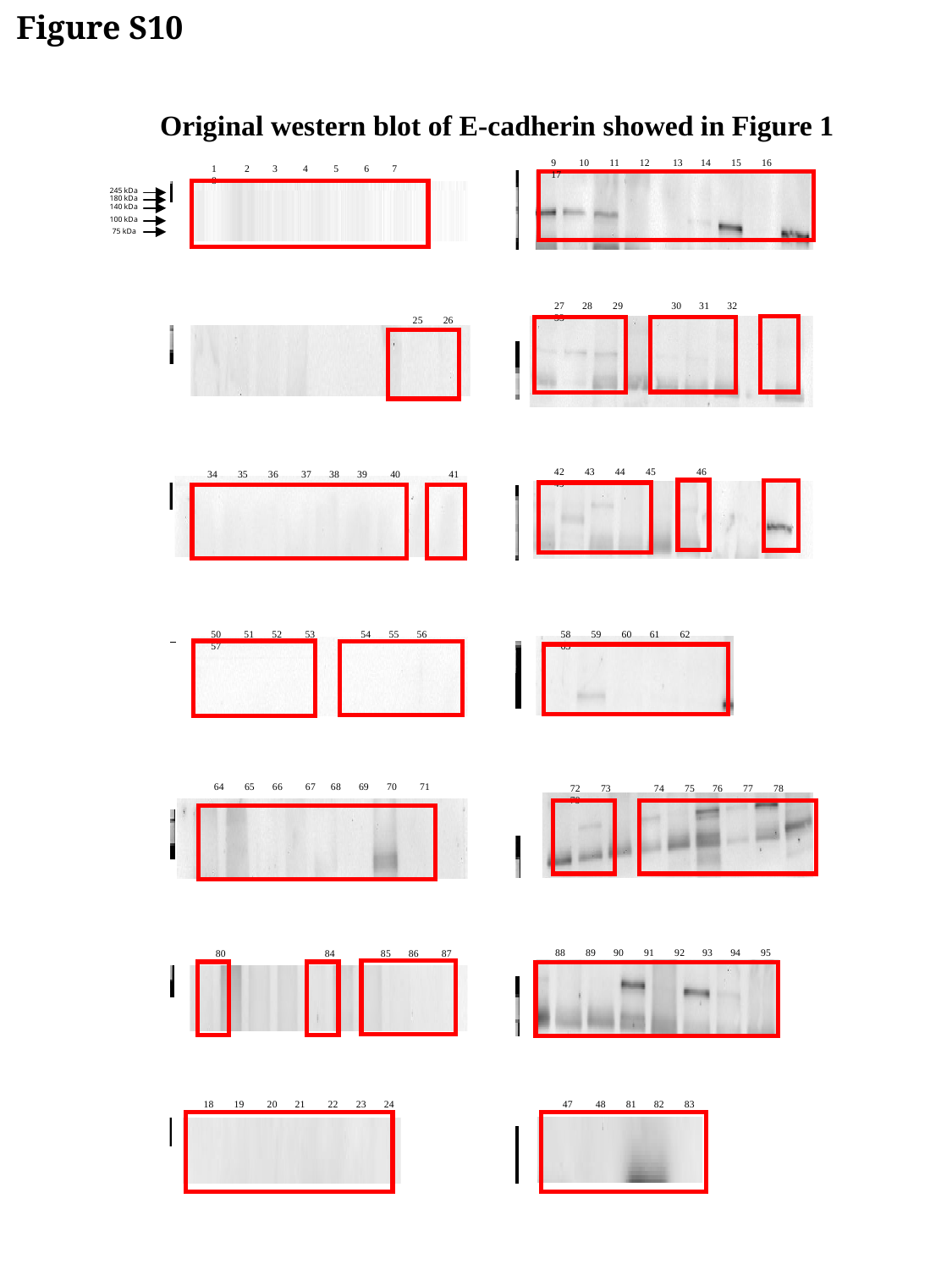

Figure S10
Original western blot of E-cadherin showed in Figure 1
9 10 11 12 13 14 15 16 17
1 2 3 4 5 6 7 8
27 28 29 30 31 32 33
25 26
42 43 44 45 46 49
34 35 36 37 38 39 40 41
50 51 52 53 54 55 56 57
58 59 60 61 62 63
64 65 66 67 68 69 70 71
72 73 74 75 76 77 78 79
88 89 90 91 92 93 94 95
80 84 85 86 87
18 19 20 21 22 23 24
 47 48 81 82 83
245 kDa
180 kDa
140 kDa
100 kDa
75 kDa

## Slide 14
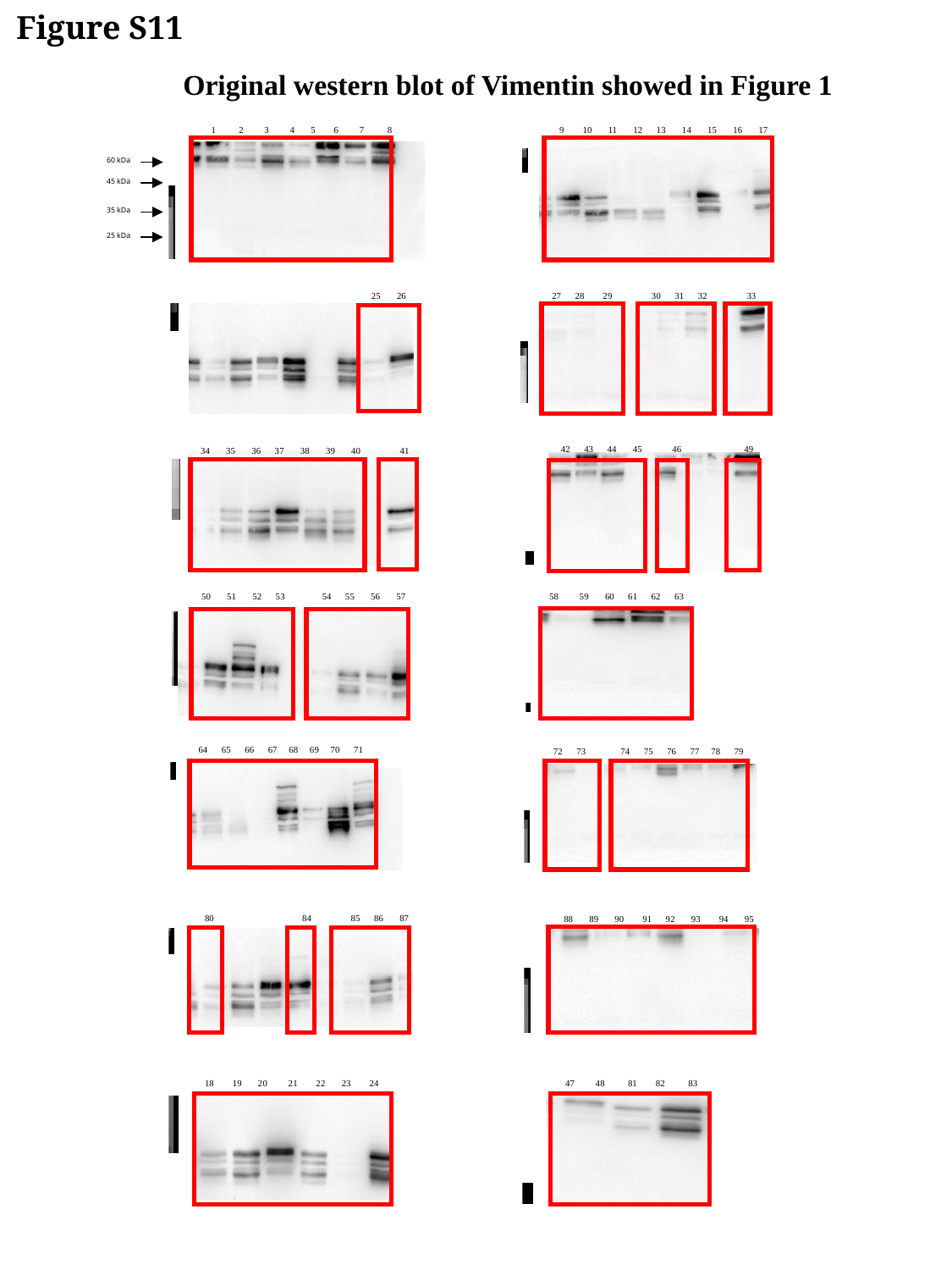

Figure S11
Original western blot of Vimentin showed in Figure 1
9 10 11 12 13 14 15 16 17
1 2 3 4 5 6 7 8
27 28 29 30 31 32 33
25 26
42 43 44 45 46 49
34 35 36 37 38 39 40 41
50 51 52 53 54 55 56 57
58 59 60 61 62 63
64 65 66 67 68 69 70 71
72 73 74 75 76 77 78 79
80 84 85 86 87
88 89 90 91 92 93 94 95
 47 48 81 82 83
18 19 20 21 22 23 24
60 kDa
45 kDa
35 kDa
25 kDa

## Slide 15
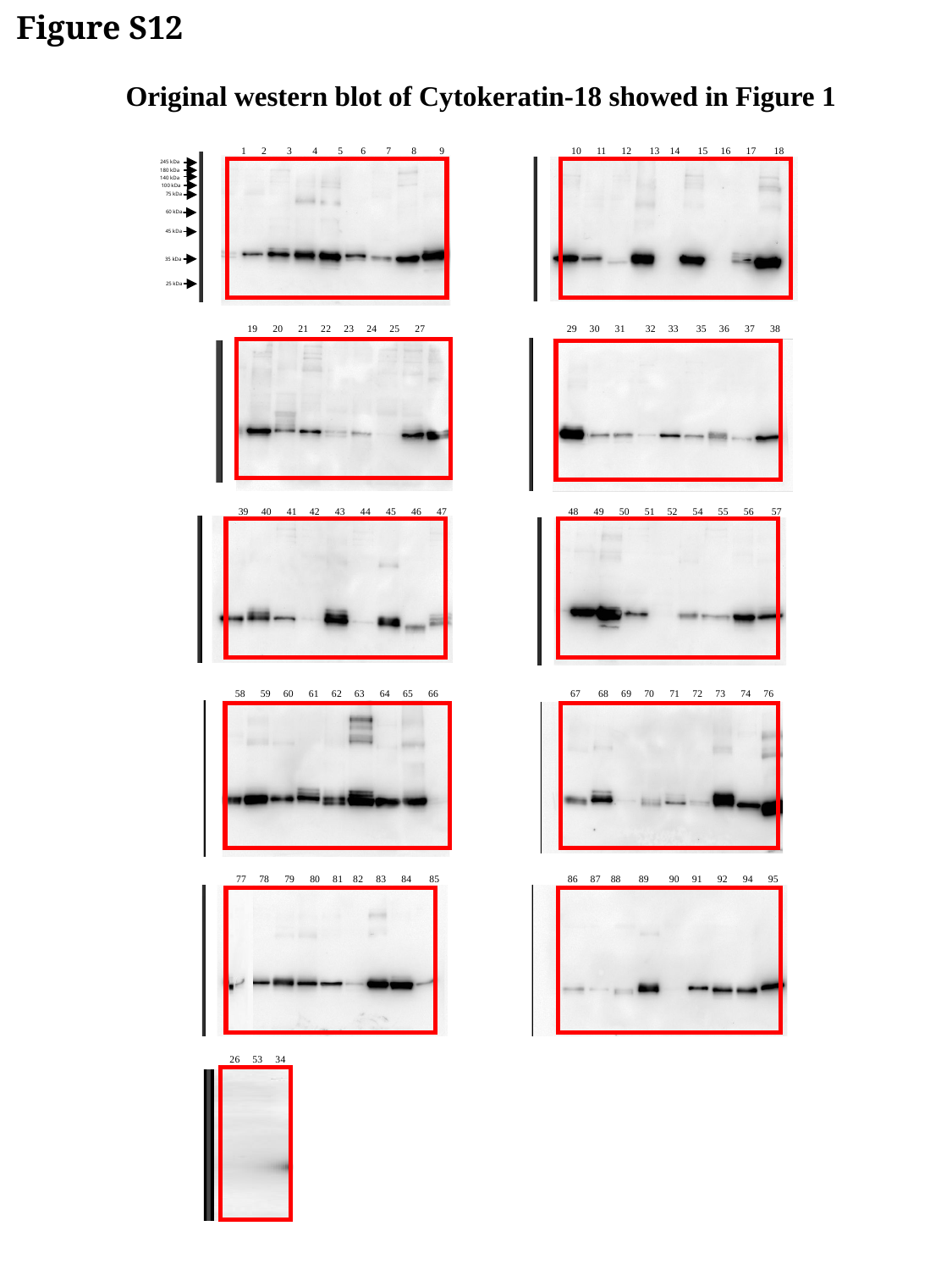

Figure S12
Original western blot of Cytokeratin-18 showed in Figure 1
1 2 3 4 5 6 7 8 9
10 11 12 13 14 15 16 17 18
245 kDa
180 kDa
140 kDa
100 kDa
75 kDa
60 kDa
45 kDa
35 kDa
25 kDa
19 20 21 22 23 24 25 27 28
29 30 31 32 33 35 36 37 38
39 40 41 42 43 44 45 46 47
48 49 50 51 52 54 55 56 57
67 68 69 70 71 72 73 74 76
58 59 60 61 62 63 64 65 66
86 87 88 89 90 91 92 94 95
77 78 79 80 81 82 83 84 85
26 53 34

## Slide 16
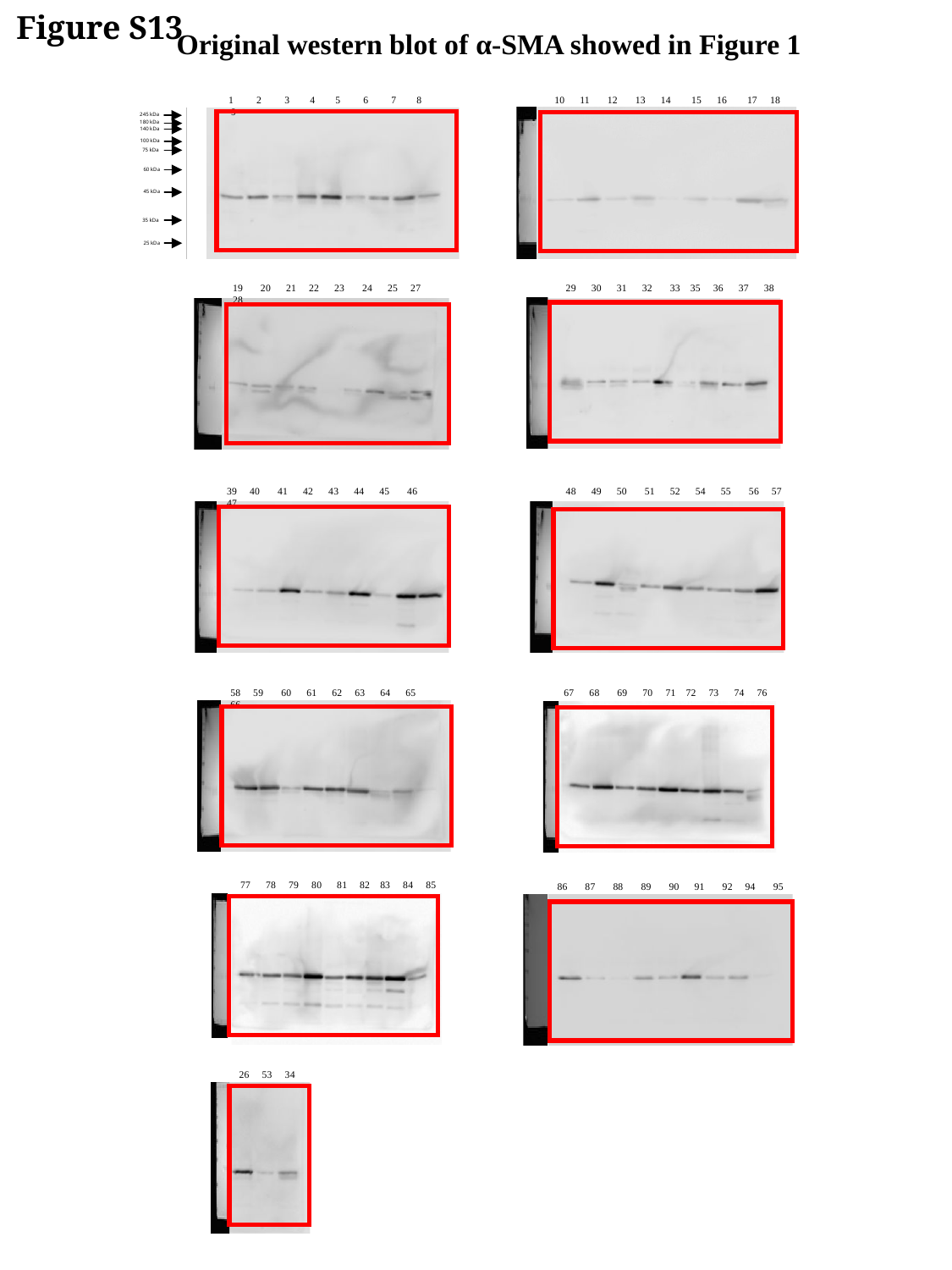

Figure S13
Original western blot of α-SMA showed in Figure 1
1 2 3 4 5 6 7 8 9
10 11 12 13 14 15 16 17 18
245 kDa
180 kDa
140 kDa
100 kDa
75 kDa
60 kDa
45 kDa
35 kDa
25 kDa
19 20 21 22 23 24 25 27 28
29 30 31 32 33 35 36 37 38
39 40 41 42 43 44 45 46 47
48 49 50 51 52 54 55 56 57
58 59 60 61 62 63 64 65 66
67 68 69 70 71 72 73 74 76
77 78 79 80 81 82 83 84 85
86 87 88 89 90 91 92 94 95
26 53 34

## Slide 17
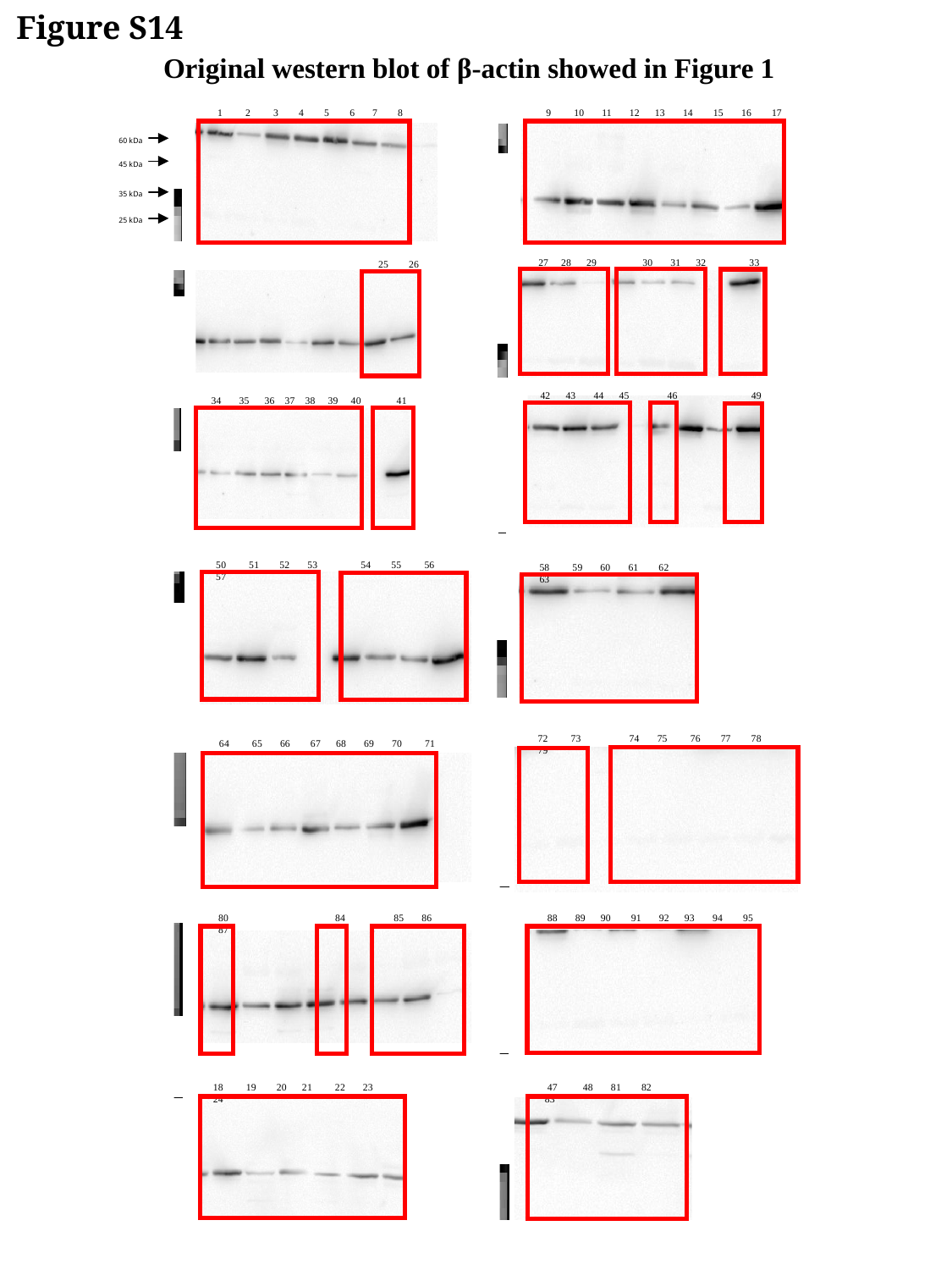

Figure S14
Original western blot of β-actin showed in Figure 1
1 2 3 4 5 6 7 8
9 10 11 12 13 14 15 16 17
60 kDa
45 kDa
35 kDa
25 kDa
27 28 29 30 31 32 33
25 26
42 43 44 45 46 49
34 35 36 37 38 39 40 41
50 51 52 53 54 55 56 57
58 59 60 61 62 63
72 73 74 75 76 77 78 79
64 65 66 67 68 69 70 71
88 89 90 91 92 93 94 95
80 84 85 86 87
 47 48 81 82 83
18 19 20 21 22 23 24

## Slide 18
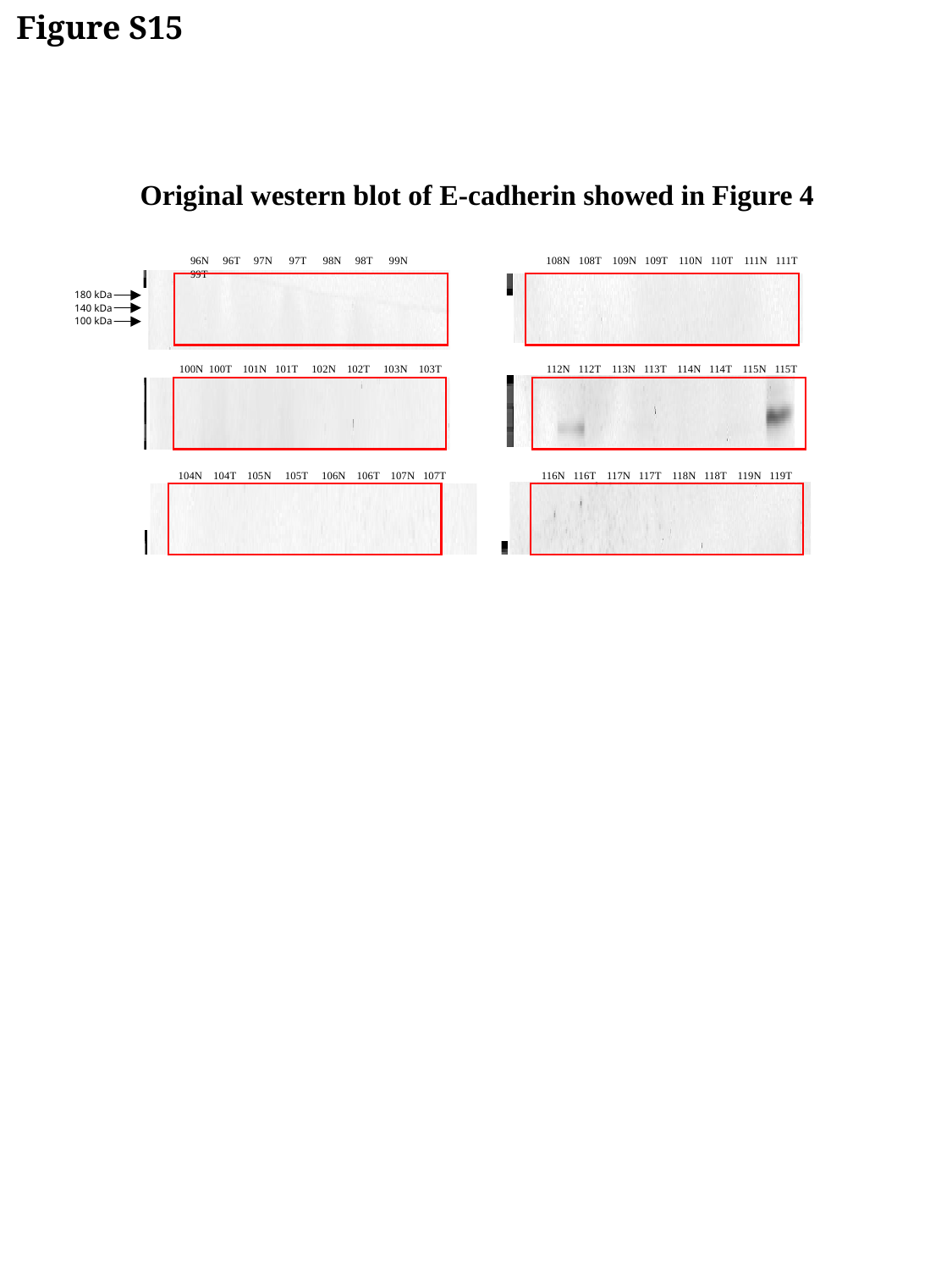

Figure S15
Original western blot of E-cadherin showed in Figure 4
96N 96T 97N 97T 98N 98T 99N 99T
108N 108T 109N 109T 110N 110T 111N 111T
180 kDa
140 kDa
100 kDa
100N 100T 101N 101T 102N 102T 103N 103T
112N 112T 113N 113T 114N 114T 115N 115T
104N 104T 105N 105T 106N 106T 107N 107T
116N 116T 117N 117T 118N 118T 119N 119T

## Slide 19
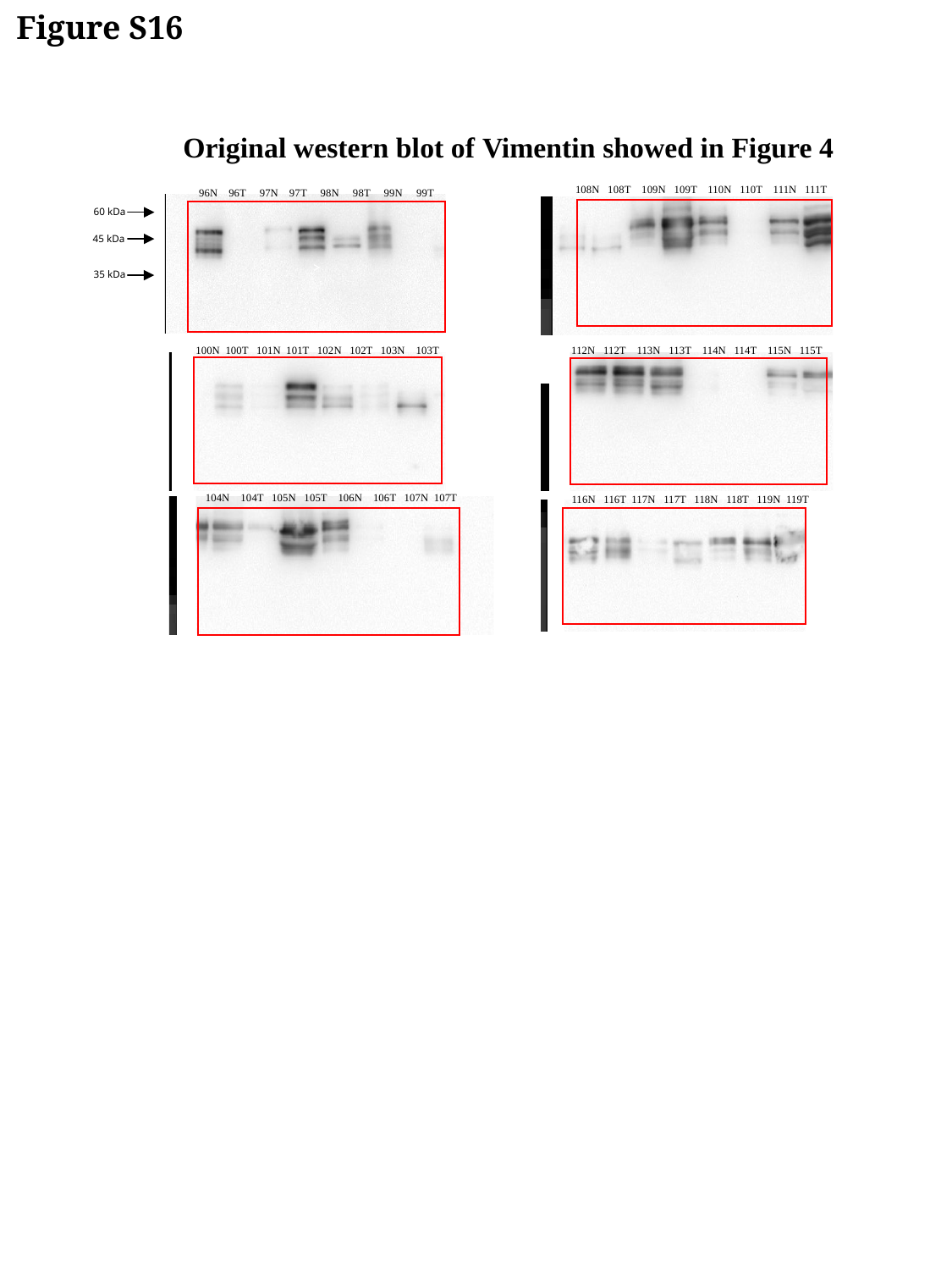

Figure S16
Original western blot of Vimentin showed in Figure 4
108N 108T 109N 109T 110N 110T 111N 111T
96N 96T 97N 97T 98N 98T 99N 99T
60 kDa
45 kDa
35 kDa
100N 100T 101N 101T 102N 102T 103N 103T
112N 112T 113N 113T 114N 114T 115N 115T
104N 104T 105N 105T 106N 106T 107N 107T
116N 116T 117N 117T 118N 118T 119N 119T
>

## Slide 20
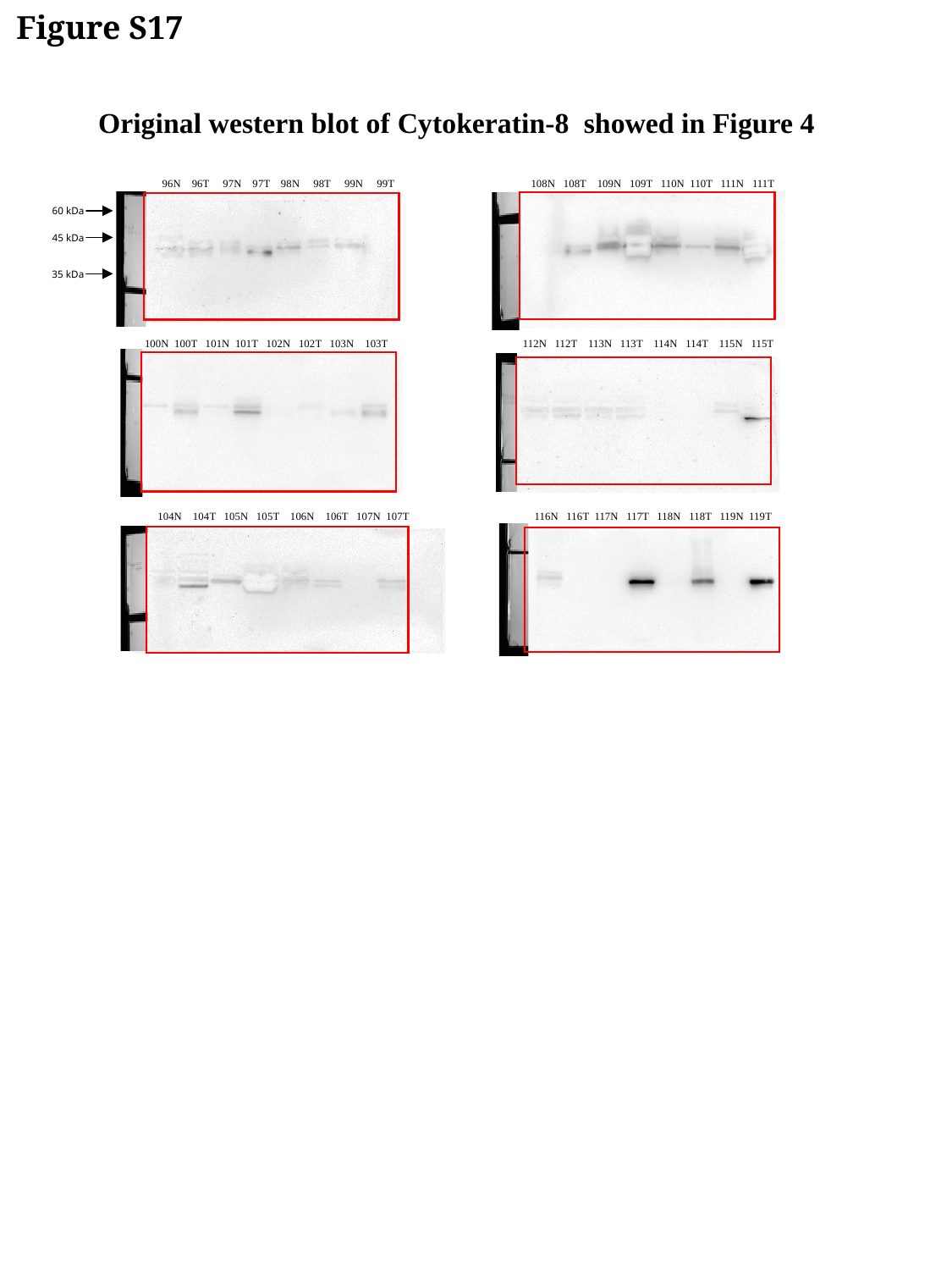

Figure S17
Original western blot of Cytokeratin-8 showed in Figure 4
96N 96T 97N 97T 98N 98T 99N 99T
108N 108T 109N 109T 110N 110T 111N 111T
60 kDa
45 kDa
35 kDa
100N 100T 101N 101T 102N 102T 103N 103T
112N 112T 113N 113T 114N 114T 115N 115T
104N 104T 105N 105T 106N 106T 107N 107T
116N 116T 117N 117T 118N 118T 119N 119T

## Slide 21
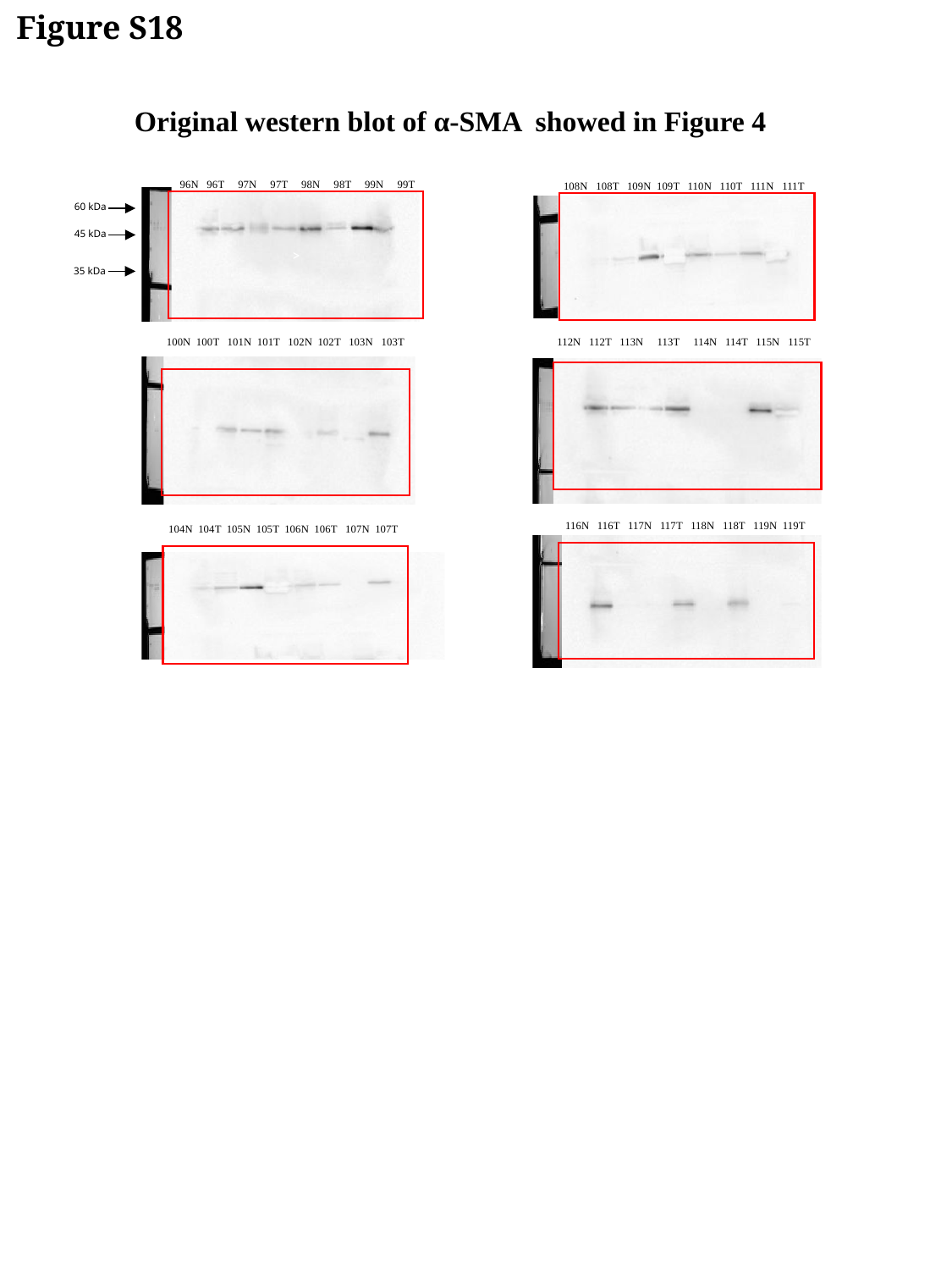

Figure S18
Original western blot of α-SMA showed in Figure 4
96N 96T 97N 97T 98N 98T 99N 99T
60 kDa
45 kDa
35 kDa
>
108N 108T 109N 109T 110N 110T 111N 111T
100N 100T 101N 101T 102N 102T 103N 103T
112N 112T 113N 113T 114N 114T 115N 115T
116N 116T 117N 117T 118N 118T 119N 119T
104N 104T 105N 105T 106N 106T 107N 107T

## Slide 22
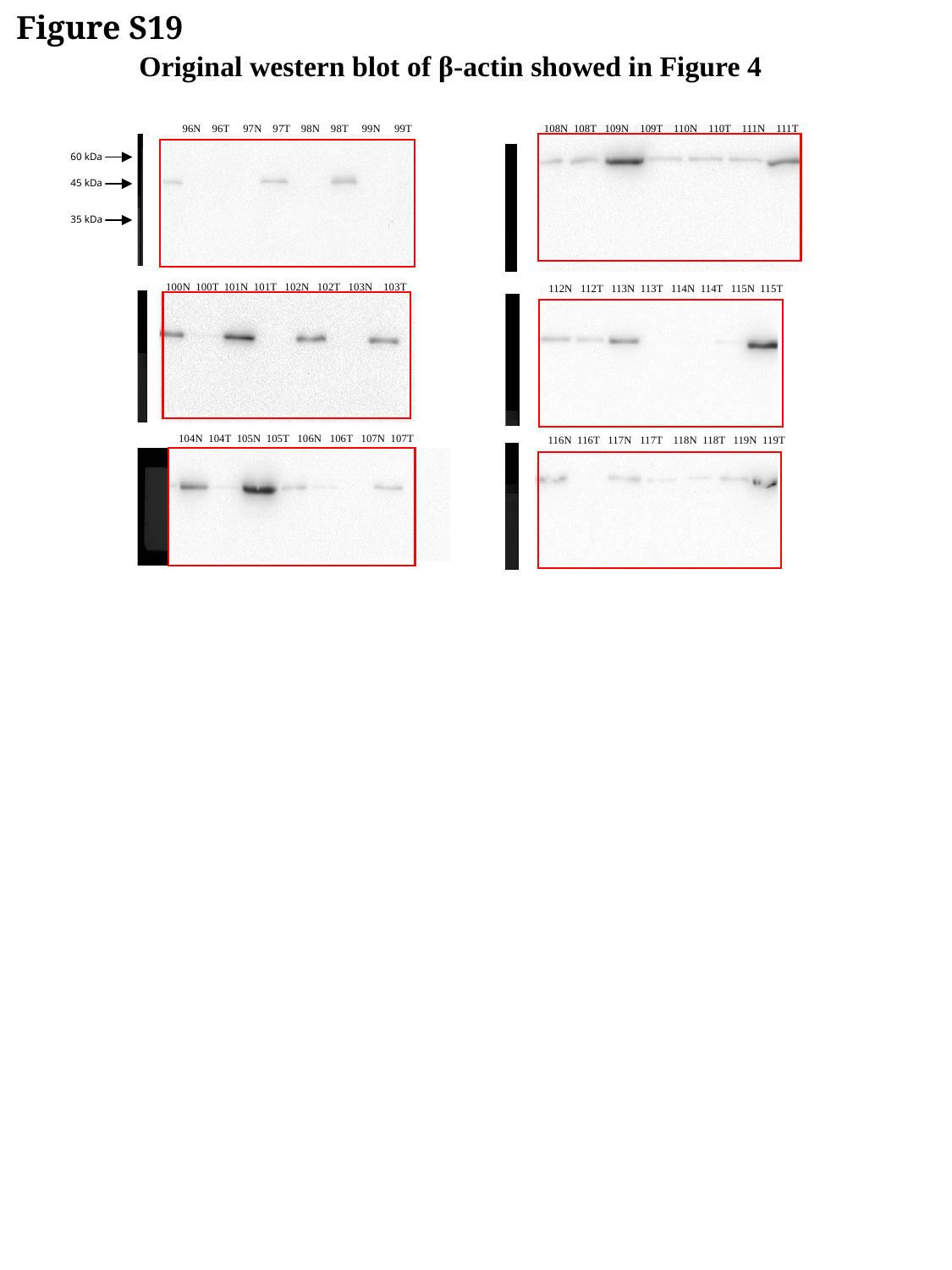

Figure S19
Original western blot of β-actin showed in Figure 4
96N 96T 97N 97T 98N 98T 99N 99T
108N 108T 109N 109T 110N 110T 111N 111T
60 kDa
45 kDa
35 kDa
100N 100T 101N 101T 102N 102T 103N 103T
112N 112T 113N 113T 114N 114T 115N 115T
104N 104T 105N 105T 106N 106T 107N 107T
116N 116T 117N 117T 118N 118T 119N 119T
>
